# The effect of mefloquine dose on outcome of uncomplicated falciparum malaria treated with artesunate-mefloquine: a WWARN systematic review and individual patient data meta-analysis

**DOI:** 10.1186/s12936-026-05963-4

**Published:** 2026-08-25

**Authors:** The WWARN AS-MQ Dose Impact Study Group, Elizabeth A. Ashley, Emmanuel Baron, Marielle K. Bouyou-Akotet, Rebekah Burrow, Gwenaelle Carn, Verena I. Carrara, Emmanuelle Comets, Chantal Csajka, Umberto D’Alessandro, Nicholas P. Day, Abdoulaye A. Djimde, Arjen Dondorp, Stephan Duparc, MAbul Faiz, Marcelo Urbano Ferreira, Lawrence Fleckenstein, Oumar Gaye, Blaise Genton, Philippe J. Guerin, Eva Maria Hodel, Georgina S. Humphreys, Peter G. Kremsner, Srivicha Krudsood, Simone Ladeia-Andrade, Gilbert Lefèvre, Rashid Mansoor, Mayfong Mayxay, Paul N. Newton, Francois Nosten, Aung Pyae Phyo, Ric N. Price, Sasithon Pukrittayakamee, Michael Ramharter, Ronnatrai Rueangweerayut, Issaka Sagara, Sodiomon B. Sirima, Frank M. Smithuis, Kasia Stepniewska, Walter R. J. Taylor, Innocent Valéa, Neena Valecha, Ingrid V. F. van den Broek, Rob W. van der Pluijm, Michèle van Vugt, Stephen A. Ward, Nicholas J. White

**Affiliations:** WorldWide Antimalarial Resistance Network (WWARN), Centre for Tropical Medicine and Global Health, University of Oxford, Oxford, UK

**Keywords:** Malaria, Artesunate, Mefloquine, Antimalarial, Meta-analysis, Efficacy

## Abstract

**Introduction:**

A combination of mefloquine associated with artesunate (AS-MQ) was the first artemisinin-based combination therapy (ACT) to be used widely for acute uncomplicated *P. falciparum* malaria.

**Methods:**

Individual patient data from 31 studies of patients with uncomplicated falciparum malaria treated with various AS-MQ regimens (target mefloquine dose 25 mg/kg), conducted in Asia, Africa and South America, were pooled and analysed to investigate the effects of MQ mg/kg dosing on malaria recurrence and other clinical, parasitological and tolerability endpoints.

**Results:**

A total of 6,761 patients were enrolled in clinical studies conducted between 1995 and 2018; the majority (74%) were from Asia. The median age of study participants was 18 years (interquartile range IQR 8–30 years), of whom 13.5% (915/6761) were aged less than 5 years old. Participants received an estimated median [range] total mg/kg dose of mefloquine and artesunate of 25 [6.8–60] and 12 [3.6–33.3] respectively, and 1,572 (23.4%) participants were treated with the coformulation.

The PCR-corrected recrudescence rate 42 days after any ASMQ treatment was 2.4% for patients enrolled in Asia and 2.5% in Africa, before artemisinin resistance emerged. Corresponding rates in children aged 1 to < 5 years were 2.9% and 3.3%. After adjusting for background artemisinin resistance, the hazard of recrudescence was higher in children aged 1 to < 5 years than in adults in Asia, but not in Africa (Asia, HR 3.01, 95%CI 1.60–5.64, p < 0.001; Africa HR 5.18, 95% CI 0.61–44.28, p = 0.133). There was only one recrudescent infection in South America. A significant MQ dose–effect was observed in Asia in patients treated with 3-dose regimens (AHR 0.89, 95% CI 0.83–0.96, p = 0.003 for 1 mg/kg increase in dose).

Vomiting rates within an hour of dosing were highest in children 1- 5 years of age (n = 140) at 3.4% doses (13/378) compared to 1.7% (20/1,168) in children 5–11 years (n = 476), and 1.1% (47/4,191) in patients 12 years of age or older (n = 2027) (p < 0.001, test for trend).

**Conclusion:**

AS-MQ administered over three days is a highly efficacious ACT in low to moderate transmission areas without artemisinin resistance. While further optimisation of the mefloquine dose in the coformulation in children aged 1–5 years could be considered, dose-related early vomiting is highest in this age group and may preclude this.

**Supplementary Information:**

The online version contains supplementary material available at 10.1186/s12936-026-05963-4.

## Background and rationale

Artesunate associated with mefloquine (AS-MQ) was the first artemisinin-based combination therapy (ACT), and was widely used in Southeast Asia from the mid-1990s onwards. Mefloquine, a fluorinated 4-quinoline methanol antimalarial, had been introduced at a dose of 15 mg/kg in combination with sulphadoxine-pyrimethamine in 1985 in Thailand but resistance emerged rapidly. This was countered temporarily by increasing the total dose to 25 mg/kg [1]. Artesunate was added to an already failing drug, in the absence of any alternative[2]. Efficacy of the loose (i.e. drugs given separately) combination exceeded the World Health Organization (WHO) target of 90% consistently in numerous studies over the next fifteen years [3]. Fixed-dose combination (FDC) antimalarials are preferred over loose combinations to facilitate treatment adherence; the AS-MQ coformulation avoids dispensing artesunate monotherapy and reduced the tablet burden from six to two tablets a day for three days for a 55 kg adult. Splitting the mefloquine dose into three doses rather than two resulted in improved mefloquine bioavailability compared to two doses [4]. The FDC was a product of the FACT (Fixed-Dose, Artesunate-Based Combination Therapies) project, launched by the Drugs for Neglected Diseases *Initiative* (DND*i*) and the UNICEF-UNDP-World Bank-WHO Special Programme for Research and Training in Tropical Diseases (TDR) in 2002, with the aim of manufacturing FDCs of AS-MQ and artesunate + amodiaquine (AS-AQ) [5]. The AS-MQ FDC was prequalified by WHO in 2012. AS-MQ was manufactured by Farmaguinhos, Brazil and latterly Cipla, India.

Millions of doses of mefloquine have been dispensed since it was introduced in 1975 and it has generally been shown to be well tolerated.[6, 7] Advantages of the combination include the long elimination half-life of mefloquine of around two weeks in patients with malaria [8, 9] and lower post-treatment gametocyte carriage compared to dihydroartemisinin-piperaquine [10]. Reports of neuropsychiatric reactions when given as prophylaxis to travellers have led to a reduction in use for this indication [11–17]. In a pooled analysis of 19,850 individual patients with malaria treated with mefloquine monotherapy or combinations in Thailand, the frequency of serious neuropsychiatric adverse events was 11.9 per 10,000 (95% CI 4 to 285) treatments following a 25 mg/kg single dose and 7.8 (3 to 15) after a 15 mg/kg dose. [18] Repeated dosing with mefloquine within a month was associated with increased risk of neurotoxicity, a barrier to use in areas of high malaria transmission [19]. Gastrointestinal adverse events e.g. diarrhoea, vomiting, abdominal pain, are common following mefloquine, particularly in young children, and are dose-related, although there is overlap with presenting symptoms of acute malaria [18].

Genetic markers of artemisinin and mefloquine resistance are *kelch13* propeller mutations [20] and multiple copy numbers of *pfmdr1*[21], respectively. Retrospective testing of samples from Cambodia has shown that *kelch13* mutations were present as early as 2001[20]. Artemisinin still retains some efficacy against parasites with *kelch13* mutations, hence the term “artemisinin partial resistance” is recommended by the WHO. The relative importance of artemisinin versus mefloquine resistance in determining efficacy of the ACT was explored in an analysis of the efficacy of AS-MQ between 2003 and 2013 along the Thai-Myanmar border [22]. Efficacy of AS-MQ was high until 2008 despite a high prevalence of multiple copy number of *pfmdr1*, but reduced significantly once artemisinin resistance became established [22].

The AS-MQ FDC is a three-day, once daily, weight or age banded treatment regimen with a target dose of 4 mg/kg/day of artesunate and 8.4 mg/kg/day of mefloquine. Before the FDC was available mefloquine was prescribed to provide a target total dose of 25 mg/kg body weight (bw) either as a single dose, or as two doses (15 mg/kg plus 10 mg/kg bw on consecutive days) or three doses (8.4 mg/kg bw daily for three days).

Individual clinical trials are typically not powered to explore differences in efficacy in patient sub-groups or between different treatment regimens. The aim of this individual patient data meta-analysis (IPD-MA) was to investigate the relationship between mefloquine dose and efficacy of AS-MQ to determine whether current dosing recommendations could be optimised further, e.g. by increasing the minimum dose of mefloquine received in some age or weight bands to improve cure rates. This systematic review and IPD-MA was registered on PROSPERO (CRD42018103776) [23] and the protocol [24] has been peer-reviewed and published separately.

## Methods

### Data acquisition

PubMed, Embase, and Web of Science databases were first searched on 29th March 2018. Full details of the search terms and screening strategy are included in Additional file 1. Studies assessing the efficacy of a three day regimen of artesunate plus mefloquine administered as a fixed dose combination or loose combination of 4 mg/kg/day artesunate plus MQ administered in two doses (15 mg/kg and 10 mg/kg on consecutive days), or three equal doses, with parasite genotyping performed for late parasitological outcome were eligible for inclusion in this meta-analysis. An update of the search, using the same screening strategy, was conducted using the WWARN Clinical Trials publication library (WCTPL) [25] of all antimalarial clinical efficacy trials conducted and published since 1946. The date of the last search was 20th April 2025. Principal investigators of the studies identified from the literature search were invited to share IPD and at least three emails were sent out in case of non-response. Researchers agreeing to the terms and conditions of the submission uploaded anonymised IPD to the WWARN repository through a secure web portal.

Raw data from individual studies were standardised using an open and transparent data management and statistical analysis protocol, and stored in a single quality assured database. Investigators were contacted for validation or clarification, if required, and individual study protocols were requested. Following identification of eligible studies, permission to include data in the current study was granted by an independent Data Access Committee, [26] or the study investigators.

### Statistical analysis

Statistical analyses were carried out using STATA (Version 17.1) according to a Statistical Analysis Plan [24]. One-stage IPD-MA was conducted to investigate the effects of MQ mg/kg dosing on (a) Polymerase Chain Reaction (PCR) genotyping-corrected risk of *P. falciparum* recrudescence (treatment failure) on day 42 (b) PCR-corrected *P. falciparum* reinfection on day 42; (c) parasite positivity on day two or day three, (d) gametocyte carriage on day seven in patients without gametocytaemia at enrolment; and (e) anaemia (Hb < 10 g/dL or Hb < 7 g/dL for severe anaemia) status on day seven. Cox regression with shared frailty (a-b) or logistic regression with random intercept (c-e) were fitted. Total mg/kg doses received were calculated from the number of tablets administered or (if not available) prescribed per protocol for each patient (Additional file 2). The effect of the following baseline covariates as potential confounders was also examined: sex, log asexual parasite density, hyperparasitaemia (asexual parasitaemia > 100,000 parasites per µL), haemoglobin (Hb), anaemia (Hb < 7 g/dL and Hb < 10 g/dL), history of fever or documented fever (> 37.5 ºC), underweight in children < 5 years of age (weight-for age z-score using WHO reference standards [27]). Models were also adjusted where appropriate for geographic region; study site malaria transmission intensity (based on *Plasmodium falciparum* prevalence (Pfpr) estimates generated by the Malaria Atlas Project (MAP) for each site latitude, longitude and the year the study was conducted: low Pfpr < 10%, moderate Pfpr 10–40%, high Pfpr ≥ 40% [28]; background artemisinin resistance status in Asia (None: 1995–1999 Cambodia + 1995–2005 other sites; Low: 2000–2004 Cambodia + 2005–2010 other sites; High: 2005–2018 Cambodia + 2011–2018 other sites [20, 29]; and individual patient parasite resistance data (*pfmdr*1 or *kelch13* markers) if available. Fractional polynomials [30] were used to explore and adjust for the nonlinear relationship with continuous variables.

Vomiting within an hour of treatment administration, any vomiting within 3 days of treatment initiation, diarrhoea within 3 days of treatment initiation, serious adverse events and neuropsychiatric adverse events were summarised. Association between MQ dose and vomiting or diarrhoea was examined using Poisson regression for the incidence, and logistic regression for vomiting after treatment administration.

Sensitivity analyses included (a) refitting final models with each study’s data excluded, one at a time, and a coefficient of variation around the parameter estimates, and (b) subset analysis including studies using at least 3 markers for PCR genotyping and patients with supervised dosing.

Risk of bias was assessed by two reviewers (EAA, KS) using the GRADE guidelines[31] and Cochrane risk of bias tool 2.0 [32]. For further details on covariates, model development, and risk of bias assessment see Additional files 1 and 3.

### Ethical approval

All data included in this analysis were obtained in accordance with the laws and ethical approvals applicable to the countries in which the studies were conducted, and were obtained with the knowledge and consent of the individual to whom they relate. Data were pseudonymised when shared with WWARN, meaning they could not be traced back to identifiable individuals. No additional ethics review was requested, in accordance with the guidance of the Oxford Central University Research Ethics Committee.

## Results

### Characteristics of included studies

**Fig. 1:**
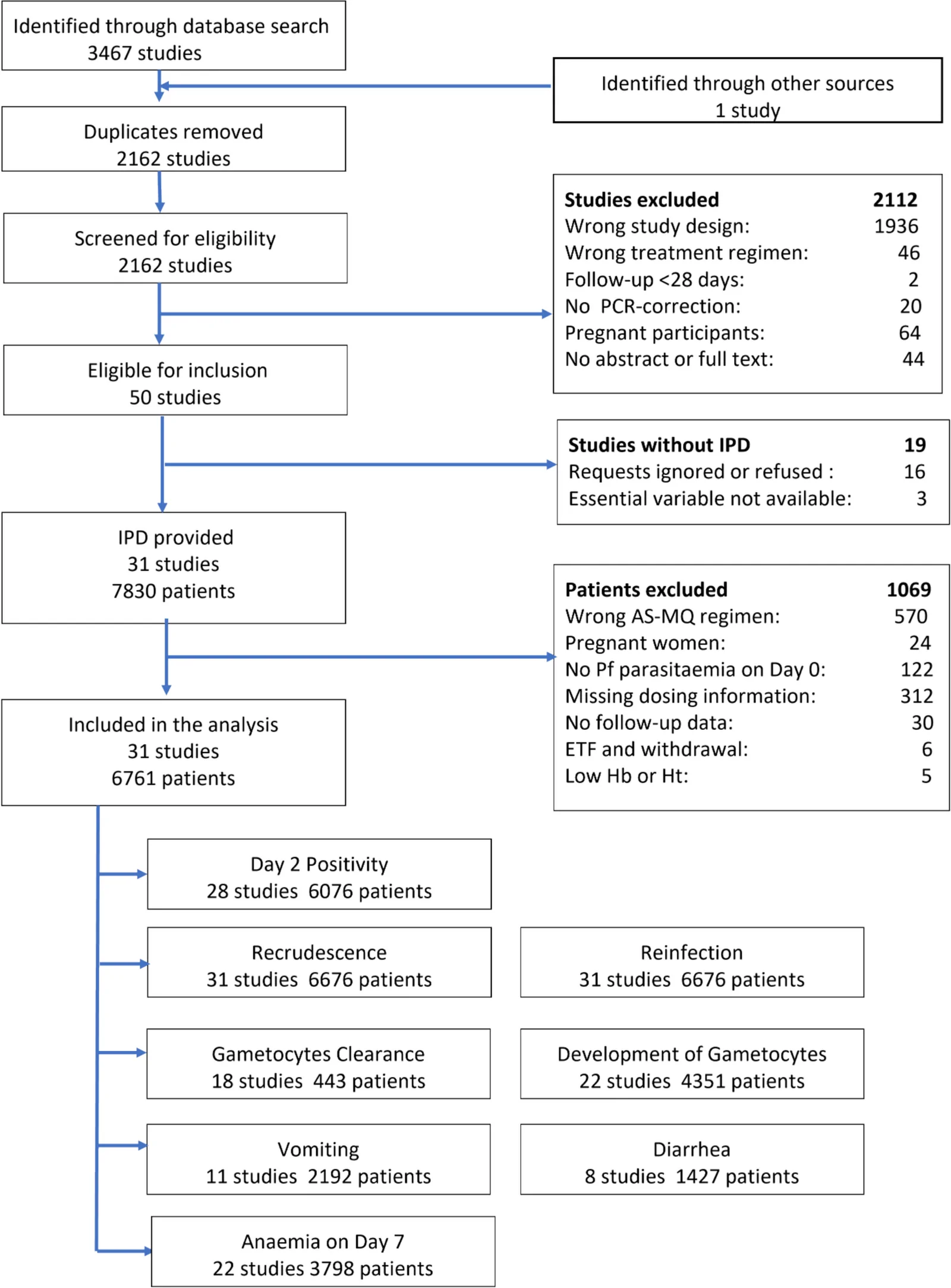
Study profile

Fifty studies were identified as eligible for inclusion and investigators of 31 of these agreed to contribute data and share individual patient records (Fig. 1; full list of studies in Additional File 2: Supplementary Table 1). Out of 7,830 patient records, 1,069 (13.7%) were excluded, leaving 6,761 patients who contributed to the analysis. Risk of bias assessment is presented in Additional file 3. Data were collected between 1995 and 2018 at 80 study sites in Asia, Africa and South America (Additional File 1: Supplementary Fig. 1). In 25% (8/31) of the studies, patients were followed-up for 28 days, in 39% (12/31) studies for 42 days, and in 35% (11/31) studies for 63 days.

### Baseline characteristics

The baseline characteristics of patients included in the analysis are presented in Table 1 showing most (74%) patients were from Asia. The majority (3,426, 50.6%) of the 6,761 participants were recruited in Thailand, followed by Myanmar (519, 7.7%), Senegal (454, 6.7%) and Cambodia (434, 6.4%).

**Table 1:** Baseline characteristics of patients included in the studies by region

Parameter	All participants		Africa		Asia		South America	
	N	Median [Range] or n (%)	N	Median [Range] or n (%)	N	Median [Range] or n (%)	N	Median [Range] or n (%)
Age (years)	6761	18 [.5–80]	1278	5 [.5–67]	5010	20 [.8–80]	473	23 [4–73]
Age group < 1 year	6761	63 (0.9)	1278	62 (4.9)	5010	1 (0.0)	473	0 (0)
1 to < 5 years	6761	852 (12.6)	1278	563 (44.1)	5010	288 (5.8)	473	1 (0.2)
5 to < 12 years	6761	1243 (18.4)	1278	331 (25.9)	5010	838 (16.7)	473	74 (15.6)
≥ 12 years	6761	4603 (68.1)	1278	322 (25.2)	5010	3883 (77.5)	473	398 (84.1)
Sex: Male	6761	4205 (62.2)	1278	645 (50.5)	5010	3276 (65,4)	473	284 (60.0)
Weight (kg)	6761	45 [5.2—110]	1278	16 [5.2—110]	5010	47 [6—85]	473	54.5 [13—96]
Underweight^a^	913	221 (24.2)	624	99 (15.9)	288	122 (42.4)	1	0 (0.0]
Haemoglobin (g/dL)	3142	11.3 [5–28]	1095	10.4 [5.2—18.9]	1577	11.6 [5—18.8]	470	12.5 [7–28]
Haematocrit (%)	5171	37 [15—69.2]	775	31.2 [15.8—62]	3925	37.7 [15—65]	471	38 [17.8—69.2]
Anaemia status^b^								
No anaemia	6210	4998 (80.5)	1095	651 (59.5)	4642	3905 (84.1)	473	442 (93.5)
Moderate anaemia	6210	1128 (18.2)	1095	398 (36.4)	4642	699 (15.1)	473	31 (6.7)
Severe anaemia	6210	84 (1.4)	1095	46 (4.2)	4642	38 (0.8)	473	0 (0.0)
Temperature (°C)	5868	37.8 [34.3—42]	1245	38.2 [34.3—41]	4150	37.6 [34.8—41.5]	473	37.8 [36–42]
Fever or history of fever^c^	5898	3807 (64.6)	1245	981 (78.8)	4180	2487 (59.5)	473	339 (71.7)
Parasitaemia (/µL)	6761	11,520 [7—469480]	1278	25,625 [18—365326]	5010	10,400 [7—469480]	473	4000 [250—64000]
Hyperparasitaemia^d^	6761	516 (7.6)	1278	163 (12.8)	5010	353 (7.1)	473	0 (0.0)
Presence of gametocytes	6300	621 (10)	1256	63 (5)	4571	455 (10)	473	103 (22)
Transmission Intensity^e^								
Low	6761	5915 (87.5)	1278	690 (54.0)	5010	5010 (100.0)	473	215 (45.5)
Moderate	6761	588 (8.7)	1278	330 (25.8)	5010	0 (0)	473	258 (54.6)
High	6761	258 (3.8)	1278	258 (20.2)	5010	0 (0)	473	0 (0)

^a^Underweight defined in children < 5 years of age, as weight-for age z-score < –2

^b^Severe anaemia defined as haemoglobin < 7 g/dL or haematocrit < 20%, moderate anaemia as 7 g/dL ≤ haemoglobin < 10 g/dL or 20% ≤ haematocrit < 30%

^c^Defined as temperature > 37·5 °C or reported history of fever

^d^Defined as parasitaemia > 10^5^/μL

^e^Malaria transmission intensity defined from estimates of *Plasmodium falciparum* prevalence rate (PfPR) according to enrolment year and location, assuming low transmission for study sites with a PfPR < 0·10, moderate transmission if PfPR 0·10 to < 0·40, and high transmission if PfPR ≥ 0·40

The median age of study participants was 18 years (interquartile range IQR 8–30 years) with 13.5% (915/6761) aged below 5 years. At enrolment, 1.4% (84/6210) of patients presented with severe anaemia (haemoglobin level < 7.0 g/dL or haematocrit < 20%), 18.2% (1,128/6,210) with moderate anaemia (7 ≤ Hb < 10 g/dL or 20 ≤ Ht < 30%), 57.8% (3,390/5,868) with fever (temperature > 37.5 °C), and 7.6% (516 /6761) had more than 100,000 parasites/µL blood (all in Africa or Asia, see Table 1); 24.2% (221/913) of the children < 5 years of age were underweight (weight-for-age z-score < -2). Overall, 87.5% (5,915/6761) of patients were recruited in low transmission areas, 8.7% (588/6761) in areas of moderate transmission and 3.8% (258/6761) in areas of high malaria transmission. In Asia, 9.9% (494/5010) were recruited at a time/region with high level artemisinin resistance, and 39.5% (1981/5010) from a time/region with a low level of artemisinin resistance.

Around half (3,255 or 48.1%) of all patients received two doses of mefloquine as loose drugs, 1,554 (23.0%) three doses of mefloquine as loose drugs, 380 (5.6%) as three doses in co-blister packaging (all in Africa) and 1,572 (23.2%) as a fixed dose combination (Table 2). Target doses of artesunate and mefloquine in the fixed dose combination are shown in Additional File 1: Supplementary Table 2.

**Table 2:** Treatment administered

	n(%) or Median [range]			
Characteristics	Africa	Asia	South America	All regions
Supervision				
Full	1278 (100.0)	4052 (80.9)	473 (100.0)	5803 (85.8)
Partial	0 (0.0)	958 (19.1)	0 (0)	958 (14.2)
Number of MQ doses				
2	0 (0.0)]	3203 (63.9)	52 (11.0)	3255 (48.1)
3	1278 (100.0)	1807 (36.1)	421 (89.0)	3506 (51.9)
Formulation				
Co-blister	380 (29.7)	0 (0.0)	0 (0.0)	380(5.6)
Fixed dose	820 (64.2)	589 (11.8)	163 (34.5)	1572 (23.4)
Loose	78 (6.1)	4421 (88.2)	310 (65.5)	4809 (71.1)
AS-MQ formulation				
Fixed dose combination, 3 MQ doses	820 (64.2)	589 (11.8)	163 (34.5)	1572 (23.3)
Loose, 2 MQ doses	0 (0.0)	3203 (63.9)	52 (11.0)	3255 (48.1)
Loose, 3 MQ doses	78 (6.1)	1218 (24.3)	258 (54.6)	1554 (23.0)
Co-blister, 3 MQ doses	380 (29.7)	0 (0.0)	0 (0.0)	380 (5.6)
Dose estimation^a^				
Tablets per protocol	1006 (78.7)	1759 (35.1)	473 (100.0)	3238 (47.9)
Tablets administered	272 (21.3)	1066 (21.3)	0 (0.0)	1338 (19.8)
Provided in study	0 (0.0)	2185 (43.6)	0 (0.0)	2185 (32.3)
Total doses received (mg/kg)				
MQ	23.1 [6.8—52.6]	25.0 [7.3 – 60.0]	23.7 [12.8 – 40.0]	25.0 [6.8 – 60]
AS	11.6 [5.5—33.3]	12.0 [3.6 – 30.0]	11.3 [6.3 – 20.0]	12 [3.6 – 33.3]
MQ (or AS-MQ) manufacturer				
Cipla	0	72 (1.4)	0	72 (1.1)
Cipla or Roche	0	981 (19.6)	0	981 (14.5)
Medochemie Ltd., Limassol, Cyprus	0	63 (1.3)	0	63 (0.9)
Far-Manguinhos	481 (37.6)	517 (10.3)	163 (34.5)	161 (17.2)
Roche	0	879 (17.5)	0	879 (13.0)
Mepha	797 (62.4)	942 (18.8)	52 (11.0)	1791 (26.5)
Atlantic Laboratories Corp., Ltd., Bangkok, Thailand	0	874 (17.5)	0	874 (12.9)
Atlantic Laboratories Corp. Ltd or Roche	0	213 (4.3)	0	213 (3.2)
Not stated	0	469 (9.4)	258 (54.6)	727 (10.8)

^a^Calculation method for the received dose

**Fig. 2:**
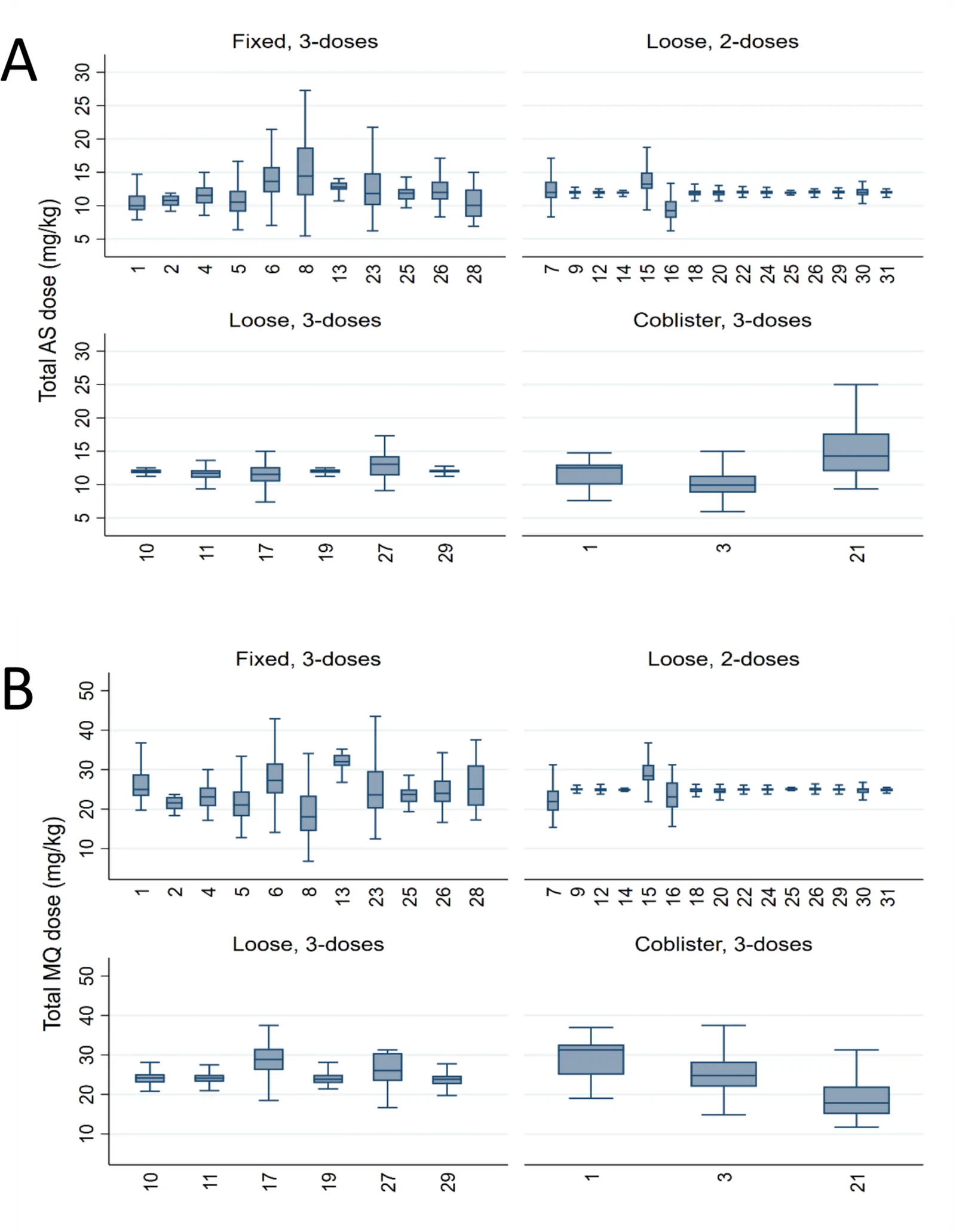
Artesunate (**A**) and mefloquine (**B**) doses administered, by study (x-axis shows study id)

Dosing was based on weight bands for 80.7% (5,459/6,761) of patients but the remaining 1,302 (19.3%) patients who were enrolled into seven studies were dosed at a specific mg dose. The mg per kg body weight doses administered (or the doses derived as per protocol if this information was not available from the submitted data) for individual studies are presented in Fig. 2, Additional File 1: Supplementary Fig. 2 and Additional file 2.

Medicine intake was fully supervised for 85.8% (5803) of patients and only the first dose was supervised for the remainder (all in Asia). Overall, the median total prescribed dose of AS was 12 mg/kg [IQR: 11.5–12.5, range: 3.6–33.3], with four patients (0.06%) administered a dose below 6 mg/kg, the recommended lowest therapeutic dose [33]. The median total prescribed dose of MQ was 25 mg/kg (IQR: 23.4–25.6, range: 6.8–60), with 793 patients (11.7%) receiving a dose less than 21 mg/kg, the WHO target minimum dose for MQ [33].

Variability in the dose administered was greater for the fixed and co-blister 3-dose regimens compared to the 2 and 3-dose loose regimens (Additional File 1: Supplementary Fig. 3), with 90% range of 21.7–30.4 mg/kg for FDC/co-blister compared to 15.0–34.7 mg/kg for the loose regimens. Patients weighing > 70 kg received substantially lower doses of AS-MQ compared to other patients (Additional File 1: Supplementary Fig. 4).

### Early parasitological response

Overall, 6,076 participants had a malaria smear on day 2 (48 h after treatment onset), of which 16.2% (986/6,076) were positive for asexual parasites. The positivity rate on day 2 varied significantly between regions (p < 0.001, adjusted for study site). In Asia 21.1% (919/4354) of patients were parasitaemic on day 2, compared to 3.8% (48/1250) in Africa and 4.0% (19/472) in South America. On day 3, 4.2% (254/6028) of patients were still parasitaemic; results by region were 5.9% (252/4304) of patients in Asia, 0.2% (2/1252) in Africa and none (0/472) in South America (see Additional File 1: Supplementary Figs. 5–6). Parasitaemia on day 2 remained similar over time in Africa and South America, but in Asia increased from 12.3% (262/2130) before artemisinin resistance emerged to 20.8% (366/1758) in areas of low resistance to 62.5% (291/466) in areas of high resistance. In Asia the corresponding day 3 positivity rates were 1.6% (34/2132), 4.3% (75/1728) and 32.2% (143/444).

Baseline parasitaemia and temperature on enrolment were positively associated with the risk of detectable parasitaemia on day 2 across all three regions, and parasitaemia on day 3 in Asia, even after adjusting for artemisinin resistance levels in Asia (Table 3; Additional File 1: Supplementary Table 3). In Asia, young age was also associated with higher risk of slide positivity on day 2 but not day 3, with highest rates observed in children 1–5 years of age.

**Table 3:** Multivariable analysis for Day 2 and Day 3 detectable parasitaemia  in Asia. Logistic regression model with random intercept for study-site and adjusted for artemisinin resistance level was fitted

	Day 2 (N = 3651, n = 774, 17 studies)			Day 3 (N = 3664, n = 213, 18 studies)		
	AOR	95% CI	p-value	AOR	95% CI	p-value
Total AS dose (mg/kg)	0.99	0.85 -1.16	0.544	0.95	0.76–1.17	0.613
Total MQ dose (mq/kg)	1.02	0.95 -1.09	0.916	1.04	0.96 – 1.12	0.324
Drug formulation*^a^						
Fixed, 3 MQ doses	0.85	0.48—1.50	0.584	0.66	0.26—1.71	0.392
Loose, 2 MQ doses	0.47	0.31—0.74	0.001	0.29	0.14—0.59	0.001
Loose, 3 MQ doses	Reference			Reference		
*Fixed, 3 MQ doses vs Loose, 2 MQ doses	1.85	1.26–2.73	0.002	2.25	1.11—4.57	0.025
Weight (kg)	1.02	1.00—1.03	0.016	1.03	1.00—1.05	0.016
Age group						
1 to < 5 years	2.72	1.41—5.25	0.003	2.12	0.58—7.81	0.258
5 to < 12 years	1.55	0.98—2.44	0.059	2.47	1.13—5.36	0.022
12+ years	Reference			Reference		
Fever or history of fever	1.81	1.43—2.30	< 0.001	1.87	1.25—2.80	0.002
log_10_ parasitaemia	2.81	2.43—3.25	< 0.001	2.95	2.25—3.86	< 0.001
Presence of gametocytes on enrolment	1.91	1.33—2.75	0.001			
Artemisinin resistance level						
None	Reference			Reference		
Low	0.77	0.56—1.07	0.117	1.19	0.65—2.2	0.563
High	2.56	1.49—4.39	0.001	5.66	2.5—13.2	< 0.001

AOR adjusted odds ratio for parasite positivity on day 2/3, *N* number of patients included, *n* number of patients with detectable parasitaemia on day 2/3

^a^Day 2 AOR 0.93, 95%CI 0.90–0.97, p = 0.001, DAY 3 AOR 0.91, 95%CI 0.85–0.98, p = 0.013 for 1 mg/kg increase in MQ dose at 24h; estimated in the same model with MQ dose at 24h included instead of drug formulation (however this model has higher AIC compared to the one presented)

There was no association between total AS or MQ dose and slide positivity on either day 2 or day 3 and this was consistent across regions (Additional File 1: Supplementary Tables 3–4), and between studies (Additional File 1: Supplementary Figs. 7–8). However, a higher MQ dose administered at 24 h was associated with a lower risk of parasitaemia on day 2 in Asia and South America, and on day 3 in Asia (Additional File 1: Supplementary Tables 3–4). After adjusting for confounding factors, the adjusted odds ratio (AOR) for a 1 mg/kg increase in MQ dose at 24 h was 0.93 (95%CI 0.90–0.97, p = 0.001) for parasitaemia on day 2 and 0.91 (95%CI 0.85–0.98, p = 0.013) for parasitaemia on day 3 (Table 3) in Asia. The corresponding rate in South America for day 2 was 0.59 (95%CI 0.37–0.85, p = 0.007). In Africa, all patients received a 3-dose MQ regimen; the variability in doses was smaller (Additional File 1: Supplementary Fig. 9) and there was no association with parasite clearance (Additional File 1: Supplementary Table 3).

There were significant differences in day 2 and day 3 slide positivity between patients treated with different formulations and this was most apparent in Asia (Table 3; Additional File 1: Supplementary Tables 3–4). In Asia, the lowest slide positivity rates were observed in patients treated with a 2-day loose MQ regimen compared to a 3-day loose MQ regimen (day 2: AOR 0.47, 95%CI 0.31—0.74, p = 0.001; day 3: AOR 0.29, 95%CI 0.14—0.59, p = 0.001), and to the 3-day fixed regimen (day 2: AOR 0.56, 95%CI 0.37–0.83, p = 0.004; day 3: AOR 0.43, 95%CI 0.21–0.88, p = 0.021).

### Treatment outcomes

Overall, 6,676 (98.7%) patients had treatment outcomes recorded. Recurrent parasitaemia could not be classified in 85 patients due to missing or indeterminate PCR results. In total, 219 (3.3%) patients had a recrudescent infection (Africa 23, Asia 195, South America 1) and 504 (7.5%) had a reinfection (Asia 296, Africa 207, South America 1). Duration of follow up varied between regions: in Africa only 3 out of 8 studies with 559 patients (out of 1278, 43.7%) followed patients beyond day 28 (to 42 or 63), compared to 18 out of 21 studies with 4859 patients (out of 5010, 96.7%) in Asia, and all 3 studies in South America.

In South America, among 473 patients, only one recrudescent and one new infection were observed so outcome data from this region were not analysed further.

### Recrudescence

The unadjusted Kaplan–Meier estimate of risk of recrudescence within 42 days was 2.4% (95%CI 1.8–3.1) for patients enrolled in Asia before artemisinin resistance, 3.5% (95%CI 2.7–4.5) for patients enrolled in Asia at a time of low artemisinin resistance, 16.1% (95%CI 12.9–19.9) for patients enrolled in Asia at a time of high artemisinin resistance, and 2.5% (95%CI 1.5–4.2) in Africa. Meta-analysis of Kaplan–Meier rates was limited as many studies did not report any recrudescences (Additional File 1: Supplementary Table 5). The risks of recrudescent malaria for individual studies are presented in Additional File 1: Supplementary Fig. 10. Time to recrudescence by region and artemisinin resistance level are shown in Additional File 1: Supplementary Fig. 11.

In both Asia and Africa, high baseline parasitaemia and high temperature at presentation were associated with greater risk of recrudescence in univariable analyses, and also in multivariable analysis, after adjusting for artemisinin resistance level in Asia (Table 4; Additional File 1: Supplementary Tables 6–8).

**Table 4:** Multivariable analysis for risk of recrudescence during 42 days follow-up. Cox regression models with shared frailty for study-site were fitted

Parameter	Asia (N = 4962, n = 185; 21 studies)			Africa (N = 1251, n = 17; 8 studies)		
	AHR	95% CI	P-value	AHR	95% CI	P-value
MQ dose (mg/kg)^a^				1.04	0.97—1.11	0.258
in 3-dose regimen	0.89	0.83—0.96	0.003			
In 2-dose regimen	1.02	0.95—1.09	0.639			
Drug formulation						
Fixed, 3 MQ doses	0.28	0.11—0.72	0.008			
Loose, 2 MQ doses	0.60	0.32—1.11	0.101			
Loose, 3 MQ doses	Reference					
log10 Parasitaemia	1.65	1.34—2.04	< 0.001			
Age						
1 to < 5 years	2.68	1.31—5.10	0.003			
5 to < 12 years	1.98	1.36—2.88	< 0.001			
12+ years	Reference					
Transmission Intensity						
High				8.94	2.40—33.30	0.001
Mod				1.95	0.46 – 8.38	0.367
Low				Reference		
Artemisinin resistance level						
None	Reference					
Low	1.09	0.64 – 1.87	0.747			
High	3.88	1.91 -7.89	< 0.001			

*AHR* adjusted hazard ratio for recrudescence by day 42, *N* number of patients included, *n* number of events observed

^a^p = 0.013 for comparison between MQ dose effects

Recrudescence rates in children aged 1 to < 5 years by day 42 were 2.9% in Asia and 3.3% in Africa before the emergence of artemisinin resistance. After adjusting for background artemisinin resistance, the hazard of recrudescence by 42 days was higher in children aged 1 to < 5 years than in adults in Asia and Africa (Asia, HR 3.01, 95%CI 1.60–5.64, p < 0.001; Africa HR 5.18, 95% CI 0.61–44.28, p = 0.133). Similar HR estimates were observed for analysis of 28 or 63 days follow-up (Additional File 1: Supplementary Tables 6–8).

There was a trend towards increasing failure rates in young children who received less than 20 mg/kg mefloquine but numbers were small and no difference compared to higher doses was demonstrated (Additional File 1: Supplementary Table 5).

In Africa, the greatest risk of recrudescence was observed in high transmission areas (day 42: HR 8.21, 95%CI 2.22–30.36, p = 0.002 compared to low transmission areas; HR 4.09, 95% CI 1.31–12.77, p = 0.015 compared to moderate transmission areas), with similar estimates in the multivariable model in which transmission intensity was a stronger predictor than the correlated variable age group (Table 4).

After adjusting for baseline parasitaemia and age, the rate of recrudescence in patients enrolled in studies before artemisinin resistance was greater in Asia compared to Africa (AHR 3.42, 95%CI 1.28- 9.17, p = 0.015). In Asia the rate of recrudescence increased over time, and was higher in studies conducted when there was low grade artemisinin resistance (AHR 3.84, 95%CI 1.44–10.29, p = 0.007), compared to no resistance, and even higher in studies conducted at the time when high grade resistance was confirmed in the region (AHR 20.41, 95% CI 7.36–56.56, p < 0.001) (Table 4).

The MQ dose–effect on the risk of recrudescence in individual studies is shown in Additional File 1: Supplementary Fig. 12. Overall, there was a significant MQ dose–effect (AHR 0.94, 95% CI 0.88–1.00, p = 0.049, for 1 mg/kg increase in dose) in Asia, but not in Africa (p = 0.256). However, the dose effect observed in Asia was due to the effect observed in patients treated with the 3-dose regimens (AHR 0.89, 95% CI 0.83–0.96, p = 0.003 for 1 mg/kg increase in dose). No MQ dose effect was observed in patients treated with 2-dose regimens (p = 0.639) (Table 4), likely because the variability in the administered dose was very small (Additional File 1: Supplementary Fig. 3). Among 3 dose regimens, similar dose effects were estimated for loose and fixed combinations (p = 0.148).

In Asia, the risks of recrudescence in patients treated with either 2 or 3 MQ doses given as loose formulations were similar (p = 0.101). However, compared to patients treated with the fixed 3 dose formulation the risk of recrudescence was higher in patients treated with the 2-dose loose formulation (AHR 2.14, 95% CI 0.95– 4.82, p = 0.065) and the loose 3 dose formulation (AHR 3.58, 95%CI 1.39–9.21, p = 0.008). Treatment supervision was not associated with recrudescence in this analysis (Additional File 1: Supplementary Tables 6–8). No interaction between mefloquine dose effect and the level of artemisinin resistance was observed in this dataset. In Africa, similarly higher recrudescence rates were observed in patients treated with the loose presentation (HR = 2.13, 95%CI 0.69–3.57, p = 0.188) compared to the fixed formulation of the 3-dose MQ regimens (Additional File 1: Supplementary Tables 6–8). Sensitivity analysis results were consistent with these findings (Additional File 1).

### Association of treatment response with molecular markers of resistance

Only three studies (ID 5,13,29) conducted molecular analysis on samples from all patients and collected data on *kelch13* and *pfmdr*1 copy number. Among these, 32.0% (287/898) infections had *kelch13* mutations in the propeller region (441-675); all in two studies in Thailand (ID 13 62/68; ID 29 225/667), and none in the study in Brazil (ID 5, 0/163).

*Kelch13* mutations were associated with increased risk of parasite positivity on day 2 (AOR 6.59, 95%CI 4.08–10.66, p < 0.001) and day 3 (AOR 10.09, 95%CI 5.63–18.08, p < 0.001) after adjusting for AS and MQ dosing and baseline parasitaemia (Table 5). However, no significant association was found between AS or MQ dose and parasite positivity on day 2 or 3. Patients treated with MQ 2-dose regimen had a significantly lower positivity rate compared to 3-dose regimens (day 2: AOR 0.52, 95%CI 0.33—0.83, p = 0.007; day 3: AOR 0.42, 95%CI 0.20—0.89, p = 0.023) after adjusting for patient-specific presence of *kelch13* mutation in the propeller region.

**Table 5:** Parasitaemia on day 2 and day 3 in studies with *kelch13* genotyping results

	AOR	95%CI	p-value
Day 2 (N = 782, n = 337, 3 studies)			
Total AS dose (mg/kg)	0.73	0.51–1.06	0.103
Total MQ dose (mg/kg)	1.02	0.81–1.28	0.885
Drug formulation			
Fixed, 3 MQ doses	2.26	0.56–9.15	0.253
Loose, 2 MQ doses	0.49	0.30–0.79	0.004
Loose, 3 MQ doses	Reference		
Log10 (parasitaemia)	2.91	2.11–4.00	< 0.001
* Kelch13* mutation (propeller region)	6.59	4.08–10.66	< 0.001
Fever	2.22	1.50–3.27	< 0.001
Region			
Asia	23.99	3.69–156.07	0.001
South America	Reference		
Day 3 (N = 739, n = 146, 3 studies)^a^			
Total AS dose (mg/kg)	0.94	0.61–1.46	0.786
Total MQ dose (mg/kg)	1.11	0.94–1.32	0.206
Drug formulation			
Fixed, 3 MQ doses	0.33	0.08–1.33	0.119
Loose, 2 MQ doses	0.37	0.17–0.78	0.009
Loose, 3 MQ doses	Reference		
Log10 (parasitaemia)	3.24	2.18–4.82	< 0.001
* Kelch13* mutation (propeller region)	10.09	5.63–18.08	< 0.001
Fever	1.88	1.15–3.07	0.011
Day 3 Asia (N = 577, n = 146, 2 studies)			
Total AS dose (mg/kg)	1.02	0.60–1.75	0.937
Total MQ dose (mg/kg)	1.01	0.85–1.21	0.900
Drug formulation			
Fixed, 3 MQ doses	1.12	0.22–5.79	0.896
Loose, 2 MQ doses	0.39	0.18–0.81	0.012
Loose, 3 MQ doses	Reference		
Log10 (parasitaemia)	3.01	2.03–4.47	< 0.001
* Kelch13* mutation (propeller region)	8.44	4.70–15.17	< 0.001
Fever	1.94	1.20–3.15	0.007

*AOR* adjusted odds ratio for parasite positivity on day 2/3, *N* number of patients included, *n* number of patients with detectable parasitaemia on day 2/3

^a^not adjusted for region, as no patients with detectable parasitaemia on day 3, so computationally not possible

Risk of recrudescence during the 42 days of follow-up was increased for malaria infections with propeller mutations (AHR 7.08, 95%CI 3.76–13.35, p < 0.001), or multiple copy number of *pfmdr1* (AHR 2.75, 95%CI 1.49–5.08, p = 0.001), and was associated with total MQ dose received (AHR 0.81, 95%CI 0.70–0.94 for 1 mg/kg increase, p = 0.007) (Table 6) However, the dataset was small and did not permit adjustment for any other covariates.

**Table 6:** Multivariable analysis for risk of recrudescence during 42 days follow-up, in studies with k*elch13* and *pfmdr1* copy number genotyping. Cox regression models with shared frailty for study-site were fitted (N = 735, n = 55)

	AHR	95%CI	P-value
Total MQ dose (mq/kg)	0.81^a^	0.70 – 0.94	0.007
*Pfmdr1* multiple copy number (> 1)	2.75	1.49–5.08	0.001
*Kelch13* mutation (propeller region)	7.08	3.76–13.35	< 0.001

It was not possible to adjust for regimen or region as failures were observed only in one study

*AHR* adjusted hazard ratio for recrudescence by day 42, *N* number of patients included, *n* number of recrudescences observed

^a^AHR (95%CI) = 0.75 (0.64–0.86), p < 0.001 for two studies in Asia (N = 572, n = 55)

### Reinfection

Analysis of reinfection rates is shown in Tables 6–8, and Additional File 1: Supplementary Figs. 13–15. The reinfections were observed between 13 and 64 days of follow-up, with an estimated 2.7% of patients experiencing reinfections by day 28, 6.5% by day 42 and 12.2% by day 63 which was the latest day of follow up in participating studies.

Similar MQ dose effect for 3-dose regimens over 42 days follow-up was observed in Africa or Asia (Table 7) and in the all regions combined model (AHR 0.97, 95%CI 0.95–1.00, p = 0.045 for 1 mg/kg increase) (Table 8). The interaction term with region was not significant (p = 0.282, likelihood ratio test). No significant dose effect was observed in the 2-dose regimen, possibly due to low variability in doses administered, as described above. For individual study estimates see Additional File 1: Supplementary Fig. 15, for univariable analysis results see Additional File 1: Supplementary Tables 9–11.

**Table 7:** Multivariable analysis for risk of reinfection during 42 days follow-up. Cox regression models with shared frailty for study-site weres fitted

Parameter	Asia (N = 4952, n = 296, 21 studies)			Africa (N = 1251, n = 207, 8 studies)		
	AHR	95% CI	P-value	AHR	95% CI	P-value
MQ dose (mg/kg)	0.98^a^	0.92–1.01	0.143	0.96	0.92 – 0.99	0.020
Sex: Male	1.45	1.04–2.00	0.026			
Weight (kg)				0.93	0.87–1.00	0.047
Age						
< 1 year				1.34	0.08–23.74	0.842^b^
1 to < 5 years	2.18	0.92–2.57	0.009	2.39	0.20 – 27.78	0.488
5 to < 12 years	1.40	0.93–1.82	0.094	1.25	0.16 -9.72	0.833
12+ years	Reference			Reference		

^a^MQ dose effect: AHR 0.92, 95%CI 0.86–0.99, p = 0.024 for 3-dose regimens; AHR 1.00, 95%CI 0.95–1.06, p = 0.898 for 2-dose regimens; p = 0.070 for interaction

^b^p = 0.028 for the likelihood ratio test comparing models with and without age group

*AHR* adjusted hazard ratio for the reinfection by day 42, *N* number of patients included, *n* number of events observed

**Table 8:** Multivariable analysis for risk of reinfection during 42 days follow-up in patients treated with 3-dose MQ regimen, all regions. Cox regression models with shared frailty for study-site were fitted

Parameter	All regions (N = 3463, n = 241, 19 studies)		
	AHR	95% CI	P-value
MQ dose (mg/kg)	0.97	0.95–1.00	0.045
Fixed combination	0.56	0.31 – 1.02	0.059
Age			
< 1 year	5.44	2.54 – 11.65	< 0.001
1 to < 5 years	6.17	3.53 – 10.77	< 0.001
5 to < 12 years	1.67	0.99 – 2.83	0.055
12+ years	Reference		
Region			
Africa	Reference		
Asia	0.72	0.38–1.39	0.328
South America	0.04	0.004 – 0.35	0.004

*AHR* adjusted hazard ratio for reinfection by day 42, *N* number of patients included, *n* number of events observed

### Gametocyte carriage

At baseline, 9.9% (621/6300) patients presented with gametocytes detected by microscopy; among those examined 23.5% were still gametocytaemic on day 7 (106/452) and 6.1% (24/394) on day 14. In Asia, gametocyte positivity at baseline increased during periods when artemisinin resistance was increasing, from 8.5% (214/2533) in times of no resistance to 11.6% (187/1607) and 12.5% (54/431) in times of low- and high-level resistance, respectively. Development of gametocytes after treatment, as evidenced by positive count on days 7 or 14 in patients without gametocytes at baseline, was observed in 1.2% (39/ 3228) of patients who did not receive primaquine and in none of the 114 individuals who received primaquine. Risk of developing gametocytes after treatment was also similar between regions with different levels of artemisinin resistance (p = 0.691). Results of more detailed analyses are presented in Additional File 1 (Supplementary Table 12–14).

### Haematological response

Among 3,798 patients from 22 studies with haemoglobin data available on day 0 and day 7, 82% (552/676) of patients who were moderately or severely anaemic at baseline were still anaemic on day 7, while 13.6% (424 /3122) of those who were not anaemic at baseline developed anaemia by day 7. Risk of anaemia on day 7 was positively associated with total mefloquine dose, mg dose at 24 h, mg dose at 48 h, or total dose over first 24 h (Additional File 1: Supplementary Table 15). In the multivariable model, both MQ and artesunate dose were independently associated with the risk of anaemia on day 7. The best fit was obtained with MQ dose at 48 h (AOR 1.08, 95%CI 1.02–1.14, p = 0.005 for 1 mg/kg increase) (Table 9). This dose–response relationship remained the same for those with and without anaemia at baseline (p = 0.868 test for interaction). Similar effect size was estimated for artesunate dose (AOR 1.07, 95%CI 1.01–1.14, p = 0.028 for 1 mg/kg increase).

**Table 9:** Multivariable analysis for moderate or severe anaemia on day 7. Logistic regression with random effect for study site was fitted

Parameter	All regions (N = 3775, n = 975, 21studies)		
	AOR	95% CI	P-value
MQ dose on Day 2 (mg/kg)^a^	1.08	1.02–1.14	0.005
Total AS dose (mg/kg)	1.07	1.01–1.14	0.028
Moderate/Severe Anaemia on Day 0	16.95	13.13 – 21.90	< 0.001
Age /Sex			
< 1 year	4.18	1.30–13.45	0.017
1 to < 5 years	1.51	0.80–2.83	0.200
5 to < 12 years	1.07	0.66–1.76	0.775
12+ years, females	2.51	1.92–3.28	< 0.001
12+ years, males	Reference		
Weight (kg)	0.97	0.95–0.98	< 0.001
Log10 parasitaemia	1.51	1.31–1.73	< 0.001
Gametocytes on Day 0	2.09	1.48–2.95	< 0.001
Region			
Africa	Reference		
South America	0.41	0.21–0.82	0.012
Asia, No artemisinin resistance	0.27	0.18–0.41	< 0.001
Asia, low artemisinin resistance	0.36	0.23–0.57	< 0.001
Asia, high artemisinin resistance	0.28	0.16–0.49	< 0.001
Transmission Intensity			
High	0.39	0.16–0.95	0.038
Moderate	0.41	0.24–0.68	0.001
Low	Reference		

^a^AIC for models with different MQ dose representation: MQ dose on day 2 (presented) 2692; total MQ dose 2697; MQ dose on day 1 2696; maximum MQ daily dose 2699, total MQ dose over first 24h 2699

*AOR* adjusted odds ratio for anaemia on day 7, *N* number of patients included, *n* number of events observed

Anaemia at baseline, young age, female sex, high temperature, high baseline parasitaemia, gametocyte carriage, transmission intensity and region (Table 9; Additional File 1: Supplementary Table 15) were also associated with the increased risk of anaemia on day 7. No association was observed with artemisinin resistance level (Table 9).

### Safety profile

A total of 51 serious adverse events were reported, among which 15 were assessed as possibly related to AS-MQ administration. Nine of these were neuropsychiatric in nature (Additional File 1: Supplementary Table 16).

Vomiting of the MQ dose in the first hour after administration was reported after 1.4% of doses (80 out of 5,737 MQ doses with available data from 2,643 patients in 9 studies), 0.9% in Africa (5 out of 582 doses in 194 patients, 1 study) and 1.5% in Asia (75 out of 5,155 doses, 2449 patients, 8 studies). Early vomiting rates were highest in children 1- 5 years of age (n = 140) at 3.4% doses (13/378) compared to 1.7% (20/1,168) in children 5–11 years (n = 476), and 1.1% (47/4,191) in patients 12 years of age or older (n = 2027) (p < 0.001 test for trend, adjusted for study-site), and in 2-dose regimen 1.6% doses (65/4182) compared to 3-dose regimen 1.0% (15/1555).

Data on vomiting or diarrhoea on days 0–3 were available for 12 studies (2195/2619, 83.8% patients) and 10 studies (1430/1853, 77.2% patients), respectively. Proportions of patients with symptoms on days 0–3 by study are presented in Additional File 1: Supplementary Tables 17–18. Incidence of vomiting during the period 1–3 days (24–72 h) after treatment onset, and risk of vomiting within 24 h after dosing, adjusted for baseline vomiting, was higher in severely anaemic patients (Table 10; Additional File 1: Supplementary Tables 19–20). Maximum daily MQ dose, but not AS dose, was associated with increased incidence of vomiting (AIRR 1.04, 95% CI 1.01–1.09, p = 0.026). Patients treated with the fixed 3-dose regimen (IRR 2.98, 95%CI 1.31–6.80, p = 0.009) and patients treated with the loose 2-dose regimen (IRR 3.74, 95%CI 1.63–8.59, p = 0.002) had a higher incidence of vomiting compared to the loose 3-dose regimen (Additional File 1: Supplementary Table 19), and confirmed in studies with full supervision.

**Table 10:** Multivariable analysis for Vomiting and Diarrhoea on days 1–3

Incidence over 1–3 days			
	AIRR	95%CI	p-value
Vomiting (N = 2086, n = 451, 12 studies)			
Vomiting Day 0	2.39	1.95- 2.92	< 0.001
Severe anaemia	1.53	1.19 – 1.99	0.001
Maximum daily MQ dose (mg/kg)	1.04	1.01–1.09	0.026
Total AS dose (mg/kg)	1.02	0.98–1.08	0.333
Diarrhoea (N = 1693, n = 284, 10 studies)			
Diarrhoea Day 0	5.48	4.15–7.24	< 0.001
Maximum daily MQ dose (mg/kg)	0.98	0.92–1.04	0.482
Total AS dose (mg/kg)	0.95	0.88–1.03	0.250
Age group			
< 1 year	2.04	0.90–4.61	0.087
1–4 years	0.98	0.49–1.95	0.945
5–11 years	0.54	0.31–0.94	0.029
12+ years	Reference		

*AIRR* Adjusted incidence rate ratio for vomiting/diarrhoea, *N* number of patients included, *n* number of incidents observed (multiple per patient)

There was no association between MQ dose and incidence of diarrhoea (Table 10). The highest incidence of diarrhoea after treatment was observed in children < 1 years of age (IRR 2.47, 95%CI 1.11–5.46, p = 0.026 compared to adults), the lowest in 5–12 years old (IRR 0.52, 95%CI 0.30–0.91, p = 0.021 compared to adults). No other covariates were found to be associated with the incidence of diarrhoea (Table 10; Additional File 1: Supplementary Tables 19–20). Similar results were observed in the sensitivity analysis (Additional File 1).

## Discussion

In this IPD-MA of data from studies of AS-MQ for the treatment of uncomplicated falciparum malaria, efficacy of the combination was shown to be very high with only 203 recrudescent infections by day 42 among 6,676 malaria episodes. However, since most of these studies were conducted, the emergence of artemisinin resistance was followed by worsening mefloquine resistance in Southeast Asia, making the failure rate of the AS-MQ combination too high to continue using it, although it has now been reintroduced in Cambodia with success [22, 34]. The FDC has become progressively more difficult to source, related to low demand; however this may change in the future as resistance to other antimalarials emerges.

After adjusting for background rates of artemisinin resistance, younger age was associated with an increased hazard of treatment failure by day 42, and was highest in the 1 to < 5 years age group. The recommended daily dose of mefloquine in the FDC for the 1 to 6 year age group (body weight 9-17 kg) is 100 mg which results in a total mefloquine dose received of 17.6 to 33.3 mg/kg in this dosing band. The WHO Malaria Treatment Guidelines propose 7 mg/kg bw as the minimum target daily dose of mefloquine in the FDC which is higher than the dose received by children weighing 17 kg at currently recommended doses.

Further dose-optimisation in the 1 to 6 year age band could be considered to increase the minimum mefloquine dose received, but the trade-off is likely to be higher rates of earlier vomiting in this age group.

Treatment failure was associated with lower mefloquine dose and a two rather than a three-dose regimen in studies in Asia. Lower risk of recrudescence in patients treated with three dose regimens is explained by increased mefloquine exposure when the total dose is split into three rather than two [35]. Despite this, some leading public health bodies, including United States Centers for Disease Control and Prevention still recommend two doses [36]. The fixed dose coformulation of AS-MQ was associated with a lower risk of recrudescence in Asia while no effect of formulation was found in Africa. The increased risk of recrudescence observed in high transmission settings in Africa compared to low transmission areas raises the possibility of misclassification by genotyping, a long-standing challenge in therapeutic efficacy studies [37]. However, overall, the number of recrudescent infections in Africa was small.

Mefloquine has a long post treatment protective effect and half of all documented reinfections occurred after 42 days.

While AS-MQ was generally well tolerated, 51 serious adverse events were reported, of which 15 were assessed as possibly related to AS-MQ administration and nine were neuropsychiatric reactions. This frequency of more than two SAEs per thousand treated patients is higher than for other antimalarials in current use and supports the recommendations to avoid repeated dosing within a short (two month) interval.

Early vomiting within an hour of mefloquine administration was dose-related but infrequent, occurring after only 1.4% doses overall but was highest in children 1- 5 years of age (n = 140) at 3.4% (13/378) doses compared to older ages groups.

Artemisinin resistance has emerged in sub-Saharan Africa in recent years, with increasing reports of slow parasite clearance from multiple countries [38]. Currently most of the African continent uses artemether-lumefantrine as first line treatment of uncomplicated falciparum malaria and there have been reports of treatment failures. [39] If artemether-lumefantrine fails, AS-MQ could be an alternative treatment for some low to moderate transmission countries where redosing within 1–2 months is infrequent. There have been concerns about lumefantrine and mefloquine cross resistance since lumefantrine is an arylaminoalcohol with structural similarities to mefloquine so this would need close monitoring. Mefloquine has already been trialled as part of a triple combination with piperaquine in areas where artemisinin resistance is high with good results [40]. Currently AS-MQ is not recommended for use as intermittent preventive treatment in pregnancy due to low tolerance with repeated dosing in previous studies [41, 42].

Limitations of this analysis include the lack of precise dosing information in several studies such that doses had to be estimated from information in the protocol, the lack of associated mefloquine pharmacokinetic data and our inability to classify all malaria recurrences as recrudescences or reinfections. Most patients were recruited in Asia and more than 35% (2368) participants were studied at a single site along the Thai-Myanmar border which also limits representativeness. Other limitations were inclusion of patients whose treatment was not supervised and the lack of individual *kelch13* and *pfmdr1* genotyping data for most participants leading to the decision to set arbitrary time periods to denote low or high artemisinin resistance. These can be justified by comparison to published data from Asia, however it is acknowledged that resistance rates were not uniform across countries in the Greater Mekong SubRegion over these time periods. In addition, there may be additional genetic factors besides *pfmdr1* copy number which affect MQ susceptibility which were not accounted for in this analysis.

## Conclusion

Given as a three day regimen at recommended doses, AS-MQ fixed dose combination is an efficacious ACT with long useful therapeutic life and good post treatment protective effect in low to moderate transmission areas without artemisinin resistance. After adjusting for background artemisinin resistance, risk of recrudescence was higher in children aged 1 to < 5 years compared to adults in this pooled analysis. While further optimisation of the mefloquine dose in the 1 to 6 year dosage band of the FDC could be considered, dose-related early vomiting is highest in this age group and may preclude this.

## Supplementary Information

Additional file1 (DOCX 8529 KB)

Additional file2 (XLSX 22 KB)

Additional file3 (XLSX 19 KB)

Additional file4 (XLSX 17 KB)

## Data Availability

The database(s) supporting the conclusions of this article are available from the WorldWide Antimalarial Resistance Network (WWARN) [http://www.wwarn.org/], a controlled open access repository of antimalarial studies on reasonable request.
