# Supplementary material for: The effect of mefloquine dose on outcome of uncomplicated falciparum malaria treated with artesunate-mefloquine: a WWARN systematic review and individual patient data meta-analysis
Source: Malar J. 2026 Aug 25;25:308. doi: 10.1186/s12936-026-05963-4 (PMC13505090; doi:10.1186/s12936-026-05963-4)
Supplement: Supplementary file 1 [file 12936_2026_5963_MOESM1_ESM.docx]

**The effect of mefloquine dose on outcome of uncomplicated falciparum malaria treated with artesunate-mefloquine: a WWARN systematic review and individual patient data meta-analysis.**

The WWARN AS-MQ Dose Impact Study Group

**Supplementary Material**

[1.	Supplementary methods	4](#_Toc234909531)

[1-1.	Systematic Review of literature methods	4](#_Toc234909532)

[1-2.	Data Acquisition	5](#_Toc234909533)

[1-3.	Variable definitions	6](#_Toc234909534)

[1-4.	Definition of Endpoints	6](#_Toc234909535)

[1-5.	Model building strategy	7](#_Toc234909536)

[2.	Drug doses in the Fixed dose combination	7](#_Toc234909537)

[Supplementary Table 2: Recommended AS and MQ dose in the FDC.	7](#_Toc234909538)

[3.	Main Analysis Supplementary Tables	8](#_Toc234909539)

[Supplementary Table 3. Univariable analysis of parasite positivity on Day 2.	8](#_Toc234909540)

[Supplementary Table 4. Univariable analysis of parasite positivity on Day 3 in Asia.	10](#_Toc234909541)

[Supplementary Table 5. Kaplan Meier estimates of the proportion of patients with recrudescence by day 42.	12](#_Toc234909542)

[Supplementary Table 6. Univariable analysis of recrudescence by day 42.	14](#_Toc234909543)

[Supplementary Table 7. Univariable analysis of recrudescence by day 28.	16](#_Toc234909544)

[Supplementary Table 8. Univariable analysis of recrudescence by day 63.	18](#_Toc234909545)

[Supplementary Table 9. Univariable analysis of reinfection by day 42.	20](#_Toc234909546)

[Supplementary Table 10. Univariable analysis of reinfection by day 28.	22](#_Toc234909547)

[Supplementary Table 11. Univariable analysis of reinfection by day 63.	24](#_Toc234909548)

[Supplementary Table 12. Univariable analysis of presence of gametocytes on day 7 in patients with gametocytaemia on enrollment.	26](#_Toc234909549)

[Supplementary Table 13. Univariable analysis of presence of gametocytes on days 7-14 in patients without gametocytaemia on enrollment.	28](#_Toc234909550)

[Supplementary Table 14. Multivariable analysis for Gametocyte carriage	31](#_Toc234909551)

[Supplementary Table 15. Univariable analysis of presence of anaemia on day 7.	32](#_Toc234909552)

[Supplementary Table 16. Serious Adverse Events and neurological adverse events in patients treated with AS-MQ	34](#_Toc234909553)

[Supplementary Table 17. Proportion of patients with vomiting, days 0-3, by study	37](#_Toc234909554)

[Supplementary Table 18. Proportion of patients with diarrhoea, days 0-3, by study	37](#_Toc234909555)

[Supplementary Table 19. Univariable analysis of vomiting and diarrhoea on day 1-3.	38](#_Toc234909556)

[Supplementary Table 20. Univariable analysis of vomiting and diarrhoea during 24h post dose.	40](#_Toc234909557)

[4.	Main Analysis Supplementary Figures	43](#_Toc234909558)

[Supplementary Figure 1. Map of study sites	43](#_Toc234909559)

[Supplementary Figure 2. Artesunate (panel A) and mefloquine (panel B) doses administered by weight, by study	44](#_Toc234909560)

[Supplementary Figure 3. Artesunate (panel A) and mefloquine (panel B) doses administered, by treatment formulation	45](#_Toc234909561)

[Supplementary Figure 4. Artesunate (A) and mefloquine (B) doses administered, by weight	46](#_Toc234909562)

[Supplementary Figure 5. Day 2 positivity by study	47](#_Toc234909563)

[Supplementary Figure 6. Day3 parasite positivity by study	48](#_Toc234909564)

[Supplementary Figure 7. MQ dose by 24H effect for positivity on Day 2 by study	49](#_Toc234909565)

[Supplementary Figure 8. MQ dose effect by study for parasite positivity on Day 3 in Asia	50](#_Toc234909566)

[Supplementary Figure 9. Mefloquine dose (mg/kg) received at 24h by region. Data in Asia and South America include 2-dose regimens. Only 3-dose regimens were studied in Africa	51](#_Toc234909567)

[Supplementary Figure 10. Recrudescence by study – outcome	day 28,42,63	52](#_Toc234909568)

[Supplementary Figure 11. Kaplan-Meier estimates of proportion of patients with recrudescence by region and artemisinin resistance level	53](#_Toc234909569)

[Supplementary Figure 12. MQ dose effect for recrudescence by study	54](#_Toc234909570)

[Supplementary Figure 13. Reinfection by study	55](#_Toc234909571)

[Supplementary Figure 14. Kaplan-Meier estimate of proportion of patients with reinfection, by transmission intensity	57](#_Toc234909572)

[Supplementary Figure 15: MQ dose effect for reinfection by study	58](#_Toc234909573)

[5.	Sensitivity analysis	60](#_Toc234909574)

[Supplementary Figure 16. Estimates (95% CI) of the dose effect (as in multivariable analysis tables) after exclusion of one study at a time. Study ID of the excluded study is shown on the x-axis. All – denotes estimates from the model fitted on all data. Estimates are OR, HR, RR or IRR	61](#_Toc234909575)

[Supplementary Figure 17. Estimates (95% CI) for the comparison between different MQ regimens after exclusion of one study at a time. The comparison group is 3-dose loose regimen. Study ID of the excluded study is shown on the x-axis. All – denotes estimates from the model fitted on all data. Estimates are OR, HR, RR or IRR	62](#_Toc234909576)

[Supplementary Figure 18. Estimates (95% CI) of the dose effect ( as in multivariable analysis tables) after exclusion of one manufacturer at a time, for supervised data , and for studies using at least 3 markers for PCR genotyping. All – denotes estimates from the model fitted on all data. Estimates are OR, HR, RR or IRR	63](#_Toc234909577)

[Supplementary Figure 19. Estimates (95% CI) for the comparison between different MQ regimens after exclusion of one manufacturer at a time, for supervised data only, and for studies using at least 3 markers for PCR genotyping. All – denotes estimates from the model fitted on all data. Estimates are OR, HR, RR or IRR	64](#_Toc234909578)

[5 Supplementary References	65](#_Toc234909579)

# 1. Supplementary methods

## 1-1. Systematic Review of literature methods

**Protocol and registration**

A protocol for this systematic review has been published^1^. The systematic review was registered with PROSPERO on 10^th^ August 2018, and was last updated on 25^th^ February 2019 (registration number CRD42018103776).

**Identifying studies – information sources**

Studies were identified by searching PubMed (1966 to present), EMBASE via OVID (1974 to present), BIOSIS via Web of Science (1969 to present) and Web of Science Core Collection via Web of Science (1969 to present). The search was carried out on 31^st^ October 2018. The WWARN IPD repository was searched for additional studies.

**Identifying studies – search**

No limits were used for these searches.

PubMed

((("plasmodium falciparum"[MeSH Terms] OR ("plasmodium"[All Fields] AND "falciparum"[All Fields]) OR "plasmodium falciparum"[All Fields]) OR falciparum[All Fields]) OR ("malaria"[MeSH Terms] OR "malaria"[All Fields])) AND (((((artesunate-mefloquine[All Fields] OR artesunate/mefloquine[All Fields]) OR ASMQ[All Fields]) OR AS-MQ[All Fields]) OR AS/MQ[All Fields]) OR (("artesunate"[Supplementary Concept] OR "artesunate"[All Fields]) AND (("mefloquine"[MeSH Terms] OR "mefloquine"[All Fields]) OR MQ[All Fields])))

EMBASE

(plasmodium falciparum.mp. OR Plasmodium falciparum/ OR falciparum.mp. OR (falciparum.mp. AND (Plasmodium/ OR plasmodium.mp.)) OR malaria.mp. OR malaria/) AND (artesunate-mefloquine.mp. OR ASMQ.mp. OR AS-MQ.mp. OR ((artesunate/ OR artesunate.mp.) AND (mefloquine/ OR mefloquine.mp. OR MQ.mp.))

BIOSIS

(TS=(plasmodium falciparum) OR (TS=(falciparum AND plasmodium)) OR TS=(falciparum) OR TS=(malaria)) AND TS=(artesunate-mefloquine) OR TS=(ASMQ) OR TS=(AS-MQ) OR (TS=(artesunate) AND TS=(mefloquine OR MQ))

Web of Science Core Collection

(TS=(plasmodium falciparum) OR (TS=(falciparum AND plasmodium)) OR TS=(falciparum) OR TS=(malaria)) AND TS=(artesunate-mefloquine) OR TS=(ASMQ) OR TS=(AS-MQ) OR (TS=(artesunate) AND TS=(mefloquine OR MQ))

Study selection processes

Results were imported into EndNote and deduplication carried out automatically and manually. One reviewer screened all non-duplicate results and assigned reason for ineligibility. A second reviewer screened the results determined to be ineligible by the first reviewer. A third and fourth reviewer screened the results determined to be eligible by the first reviewer. Disagreements regarding reason for ineligibility and eligibility status were resolved by discussion.

**Study Eligibility**

**Inclusion criteria**

1. **Prospective clinical efficacy study of uncomplicated P. falciparum (either alone or mixed infections)**
2. **Assessing the efficacy of a fixed-dose ASMQ combination, either as single tablet type, or co-blister pack of more than one tablet type, or assessing the efficacy of a loose combination of ASMQ**
3. **Where AS was given over three days (with any number of doses per day) with a target total dose of 6-30mg/kg**
4. **Where MQ was given over 2-3 days, on any of days 1-3 (with any number of doses per day) with a target total dose of 15-33mg/kg, which if MQ is split over two days, must be split 10mg/kg for the first day, and 15mg/kg the second day.**
5. **Where all AS and MQ were administered orally**
6. **With a minimum of 28 days follow up**
7. With genotyping performed for late parasite recurrence where there were treatment failures after day 7 (D7), and when patients were not retained in hospital for 28 days of follow-up.
8. **With individual patient data on dosage of mefloquine received (actual or per protocol) by patients (dosage per tablets, number of tablets given per dose and duration of treatment)**

**Exclusion criteria**

9. **Where other antimalarial drugs were given in addition to the initial ASMQ treatment regimen, except for a single dose of primaquine of 0.25mg/kg in the first 3 days**
10. **Where all participants are pregnant**
11. **Where publication states that follow-up visits are less often than weekly**

**Participant Eligibility**

**Exclusion criteria**

12. **Pregnant**
13. **Received other antimalarial drugs during follow-up before recorded P. falciparum treatment failure**
14. **Results of genotyping performed for late parasitological outcome are not available**
15. **No or missing *Plasmodium falciparum* infection on enrolment**
16. **Data on age and (or) weight and (or) gender are missing**
17. **Any of these other deviations, as defined by Clinical Module DMSAP [1]:**
a. **Haemoglobin < 5 g/dL on day 0**
b. **Haematocrit < 15% on day 0**

## 1-2. Data Acquisition

Investigators of studies satisfying the inclusion criteria were invited to participate in this IPD meta-analysis. Individual study protocols were available for all trials included, either from the publication or as a metafile submitted with the raw data. Individual patient data from eligible studies were previously shared with WWARN, collated and standardised using previously described methodology.^1^ Analysis was conducted on data extracted as of 31 May 2024.

## 1-3. Variable definitions

The doses of artemisinin and mefloquine received were calculated from the number of daily tablets administered to each patient. If the daily tablet counts were not available, doses were back-calculated using the dosing scheme available from study protocols.

The study sites were classified into 3 categories of *Plasmodium falciparum* malaria transmission intensity: low, medium and high malaria based on the *Plasmodium falciparum* parasite prevalence (PfPR) estimates obtained from the Malaria Atlas Project for specific location and year of study (for methodology see ^2^). Study sites with a PfPR <0·10 were classified as low, with PfPR from 0·10 to <0·40 as moderate and with PfPR ≥0·40 as high transmission areas.^3^

Nutritional status was assessed in children < 5 years of age with standardised age, weight, height and gender specific growth reference according to the WHO 2006 recommendations using zscore06 stata command. The nutritional status of a child was given as a Z-score and classified as underweight (WAZ<-2), as defined in the WHO guidelines.^4, 5^

In patients with missing haemoglobin concentration, haematocrit was converted to haemoglobin using the following relationship^6^:

$Hematocrit \left( ht \right)=5\cdot62+2\cdot60 \times Haemoglobin (hb)$

Anaemia was defined as severe when haemoglobin concentration was below 7g/dl and as moderate if haemoglobin concentration was between 7-10g/dL.

## 1-4. Definition of Endpoints

a) ***Early Parasitological response***

A pre-defined algorithm was used to estimate positivity status on days 2 or 3, if no observation of the blood film was recorded on that day as described in WWARN clinical Module DMSAP ^1^. For studies with frequent or irregular sampling, a patient was classified as being positive on days 1, 2 and 3 after enrolment if the measurements within a window of ±2 hours of 24, 48 and 72 hours were positive.

b) ***Plasmodium falciparum recrudescence***

Recrudescence was defined as recurrence of asexual *Plasmodium falciparum* (Pf) parasitaemia of the same genotype(s) that caused the original illness, due to incomplete clearance of asexual parasites after antimalarial treatment.

For the analysis of *Plasmodium falciparum* recrudescence, event was defined as Polymerase Chain Reaction (PCR) confirmed Pf recrudescence. Patients who had a new infection (of any species) were censored at the first day the positive parasitaemia was recorded. Patients who had a reappearance of Pf parasites but the PCR result was not available were excluded from the analysis to avoid informative censoring.

c) ***Plasmodium falciparum re-infection***

Reinfection was defined as a new Pf infection that follows a primary infection; distinguished from recrudescence by the parasite genotype, which is often (but not always) different from that which caused the initial infection.

For the analysis of *Plasmodium falciparum* re-infection, failure were defined as PCR confirmed Pf re-infection. Patients who had a new infection other than Pf species or Pf recrudescence were censored at the first day the positive parasitaemia was recorded. Patients who had a reappearance of Pf parasites but the PCR result was available were excluded from the analysis to avoid informative censoring.

## 1-5. Model building strategy

For all multivariable analyses the same approach was used. The WHZ score, age category and ACT treatment were kept in the model regardless of their statistical significance. The remaining covariates were investigated for inclusion in the model using a general strategy recommended by Collett.^7^

i) All possible risk factors were examined in a univariable analysis. The log-likelihood estimates $(-2\times Log\hat{L})$ were compared against the null model to assess if any of the variables reduce its value at 5% level of statistical significance.
ii) All the variables identified in step (i) were fitted together in one model and variables which were not significant in the presence of other variables based on the results of the Wald test were identified.
iii) A likelihood ratio test was used to assess the impact of omitting variables identified in step (ii). If the omitted variable did not significantly impact the model log-likelihood, then they were dropped. Only variables which led to significant change in log-likelihood were retained.
iv) All variables excluded from step (i) were added to the model identified in step (iii) one by one to check if they provided any improvement to the model.
v) A final check of the model identified in step (iv) was carried out to ensure that none of the variables in the model could be omitted without significantly increasing the model log-likelihood, and none of the excluded variables significantly reduced the model log-likelihood.
vi) In the final model identified in v) interaction term between ACT and WHZ was added and likelihood ratio test was used to assess the improvement to the model.

The underlying assumption of proportional hazards was tested in the final model using Schenfoeld residuals and reported when violated. For variables which violated the assumption, interaction between this variable and time intervals (0-28, 28-42 days) were considered for inclusion in the final model.

**Fractional polynomials^8^ were used to adjust for the nonlinear relationship with continuous variables.**

# 2. Drug doses in the Fixed dose combination

## Supplementary Table 2: Recommended AS and MQ dose in the FDC.

| **Weight (kg)** | **Age** | **Recommended dose** | **AS total mg/kg** | **MQ total mg/kg** |
| --- | --- | --- | --- | --- |
| 5-8 | 6 to 11 months | One tablet 25/55 mg daily for 3 days | 9.4 to 15 | 18.8 to 30 |
| 9-17 | 1 to 6 years | Two tablets 25/55 mg daily for 3 days | 8.8 to 16.7 | 17.6 to 33.3 |
| 18-29 | 7 to 12 years | One tablet 100/220 mg daily for 3 days | 10.3 to 16.7 | 20.7 to 33.3 |
| >30 | >13 years | Two tablets 100/220 mg daily for 3 days | < 9.2 (if bw >65kg) to 20 | <18.5 (if bw >65kg) to 40 |

AS/MQ doses are shown in two different tablet strengths. Mefloquine HCl 55mg and 220 mg are equivalent to 50mg and 200mg mefloquine respectively

# 3. Main Analysis Supplementary Tables

## Supplementary Table 3. Univariable analysis of parasite positivity on Day 2.

Logistic regression models with random intercept models for study-site were fitted. Models for Asia are adjusted for artemisinin resistance level.

|  | Asia | | | | Africa | | | | South America | | | |
| --- | --- | --- | --- | --- | --- | --- | --- | --- | --- | --- | --- | --- |
|  | N | % | OR (^9^5% CI) | P-value | N | % | OR (95% CI) | P-value | N | % | OR (95% CI) | P-value |
| Number of MQ doses |  |  |  |  |  |  |  |  |  |  |  |  |
| Three | 1579 | 36.9 | 2.05 (1.57 - 2.69) | <0.001 | 1250 | 3.8 |  |  | 420 | 4.5 |  | 0.250* |
| Two | 2775 | 12.1 | Reference |  |  |  |  |  | 52 | 0 |  |  |
| MQ dose (mg/kg) at 24h | 4354 | 21.1 | 0.93 (0.90 - 0.96) | <0.001 | 1250 | 3.8 | 1.00 (0.86 - 1.15) | 0.946 | 472 | 4.0 | 0.67 (0.47 - 0.95) | 0.025 |
| Total MQ dose (mg/kg) over first 24h | 4354 | 21.1 | 1.02 (0.98 - 1.07) | 0.277 | 1250 | 3.8 | 1.00 (0.93 - 1.07) | 0.946 | 472 | 4.0 | 0.87 (0.72 - 1.06) | 0.172 |
| Total AS dose (mg/kg) | 4354 | 21.1 | 1.02 (0.95 - 1.09) | 0.636 | 1250 | 3.8 | 1.00 (0.92 - 1.09) | 0.926 | 472 | 4.0 | 0.89 (0.69 - 1.15) | 0.363 |
| Drug formulation |  |  |  |  |  |  |  |  |  |  |  |  |
| Fixed, 3 MQ doses | 415 | 39.5 | 0.76 (0.45 - 1.27) ^1^ | 0.287 | 796 | 5.2 | 4.13 (0.56 – 30.42)^2^ | 0.164 | 162 | 5.6 | 1.46 (0.58 - 3.67) | 0.423 |
| Loose, 2 MQ doses | 2775 | 12.1 | 0.42 (0.29 - 0.62) | <0.001 | 0 |  |  |  | 52 | 0 |  | 0.223* |
| Loose, 3 MQ doses | 1164 | 35.9 | Reference |  | 77 | 1.3 | Reference |  | 258 | 3.9 | Reference |  |
| Co-blister, 3 MQ doses | 0 |  |  |  | 377 | 1.6 | 1.23 (0.15 – 10.36) | 0.850 | 0 |  |  |  |
| Supervision |  |  |  |  |  |  |  |  |  |  |  |  |
| Partial | 605 | 27.9 | 1.27 (0.37 - 4.31) | 0.702 |  |  |  |  |  |  |  |  |
| Full | 3749 | 20.0 |  |  |  |  |  |  |  |  |  |  |
| Age (years) | 4354 | 21.1 | 0.99 (0.98 - 1.00) | 0.001 | 1250 | 3.8 | 0.98 (0.94 - 1.02) | 0.354 | 472 | 4.0 | 1.01 (0.98 - 1.04) | 0.411 |
| Age group |  |  |  |  |  |  |  |  |  |  |  |  |
| < 1 year | 0 |  |  |  | 61 | 6.6 | 2.53 (0.69 - 9.28) | 0.163 | 0 |  |  |  |
| 1 to <5 years | 203 | 17.2 | 1.82 (1.17 - 2.84) | 0.008 | 562 | 4.4 | 1.72 (0.71 - 4.20) | 0.233 | 0 |  |  |  |
| 5 to <12 years | 622 | 18.2 | 1.33 (1.03 - 1.73) | 0.031 | 331 | 3.3 | 1.27 (0.48 - 3.38) | 0.635 | 74 | 4.1 | 1.01 (0.29 - 3.54) | 0.992 |
| ≥ 12 years | 3528 | 21.8 | Reference |  | 296 | 2.7 | Reference |  | 397 | 4.0 | Reference |  |
| Underweight (<5 years of age)^3^ |  |  |  |  |  |  |  |  |  |  |  |  |
| Yes | 67 | 7.5 | 0.31 (0.09 – 1.09) | 0.067 | 98 | 2.0 | 0.38 (0.09 -1.64) |  | 0 |  |  |  |
| No | 136 | 22.8 | Reference |  | 524 | 5.2 | Reference |  | 0 |  |  |  |
| Sex |  |  |  |  |  |  |  |  |  |  |  |  |
| Male | 2854 | 22.1 | 1.21 (0.99 - 1.47) | 0.062 | 643 | 3.4 | 0.78 (0.44 - 1.40) | 0.412 | 284 | 4.9 | 1.90 (0.67 - 5.36) | 0.226 |
| Female | 1500 | 19.1 | Reference |  | 607 | 4.3 | Reference |  | 188 | 2.7 | Reference |  |
| Haemoglobin (g/dL)^4^ | 4015 | 21.1 | 1.03 (0.99 - 1.07) | 0.122 | 1068 | 3.7 | 0.89 (0.73 - 1.07) | 0.213 | 472 | 4.0 | 0.95 (0.74 - 1.23) | 0.704 |
| Moderate/Severe anaemia^5^ |  |  |  |  |  |  |  |  |  |  |  |  |
| Yes | 490 | 14.7 | 0.94 (0.70 - 1.27) | 0.687 | 439 | 4.1 | 0.98 (0.49 - 2.00) | 0.965 | 31 | 3.2 | 0.78 (0.10 - 6.07) | 0.815 |
| No | 3525 | 22.0 | Reference |  | 629 | 3.3 | Reference |  | 441 | 4.1 | Reference |  |
| Severe anaemia^6^ |  |  |  |  |  |  |  |  |  |  |  |  |
| Yes | 22 | 9.1 | 0.69 (0.13 - 3.62) | 0.661 | 46 | 6.5 | 1.56 (0.45 - 5.44) | 0.484 | 0 |  |  |  |
| No | 3993 | 21.2 | Reference |  | 1022 | 3.5 | Reference |  | 472 | 4.0 |  |  |
| Temperature (C) | 3690 | 21.2 | 1.66 (1.52 - 1.82) | <0.001 | 1218 | 3.9 | 1.51 (1.11 - 2.05) | 0.009 | 472 | 4.0 | 1.49 (0.95 - 2.34) | 0.085 |
| Fever or history of fever ^7^ |  |  |  |  |  |  |  |  |  |  |  |  |
| Yes | 2247 | 25.2 | 2.29 (1.83 - 2.87) | <0.001 | 975 | 4.3 | 1.97 (0.80 - 4.82) | 0.139 | 338 | 4.4 | 1.51 (0.49 - 4.63) | 0.472 |
| No | 1469 | 15.0 | Reference |  | 243 | 2.5 | Reference |  | 134 | 3.0 | Reference |  |
| Log10 Parasitaemia (/µL) | 4354 | 21.1 | 3.00 (2.63 - 3.44) | <0.001 | 1250 | 3.8 | 3.22 (1.73 - 6.00) | <0.001 | 472 | 4.0 | 3.98 (1.41 - 11.25) | 0.009 |
| Hyperparasitaemia ^8^ |  |  |  |  |  |  |  |  |  |  |  |  |
| Yes | 341 | 42.8 | 4.39 (3.30 - 5.85) | <0.001 | 162 | 8.6 | 2.93 (1.49 - 5.77) | 0.002 | 0 |  |  |  |
| No | 4013 | 19.3 | Reference |  | 1088 | 3.1 | Reference |  | 472 | 4.0 |  |  |
| Gametocytes on enrolment |  |  |  |  |  |  |  |  |  |  |  |  |
| Yes | 285 | 30.2 | 1.32 (0.97 - 1.82) | 0.082 | 63 | 1.6 | 0.38 (0.05 - 2.81) | 0.342 | 103 | 4.9 | 1.29 (0.45 - 3.68) | 0.629 |
| No | 3630 | 21.1 | Reference |  | 1187 | 4.0 | Reference |  | 369 | 3.8 | Reference |  |
| Transmission Intensity^9^ |  |  |  |  |  |  |  |  |  |  |  |  |
| High | 0 |  |  |  | 258 | 2.7 | 0.77 (0.27 - 2.24) | 0.636 | 0 |  |  |  |
| Moderate | 0 |  |  |  | 306 | 4.6 | 1.18 (0.54 - 2.54) | 0.681 | 258 | 3.9 | 0.92 (0.37 - 2.30) | 0.856 |
| Low | 4354 | 21.1 |  |  | 686 | 3.9 | Reference |  | 214 | 4.2 | Reference |  |

^1^comparison between Fixed, 3 MQ doses and Loose, 2 MQ doses: OR (95% CI)=1.69 (1.17-2.44), p=0.005.,^2^ comparison between Fixed, 3 MQ doses and Loose, combined: OR (95% CI)=3.47 (1.54-7.69), p=0.003; ^3^Underweight defined in children <5 years of age, as weight-for age z-score <–2;^4^Ht converted to Hb using the formula $Hematocrit =5\cdot62+2\cdot60 \times Haemoglobin$ (Lee, 2008); ^5^Severe anaemia defined as haemoglobin <7 g/dL or haematocrit<20%; ^6^Moderate anaemia as 7 g/dL ≤haemoglobin <10 g/dL or 20%≤haematocrit<30%; ^7^Defined as temperature >37·5°C or reported history of fever; ^8^Defined as parasitaemia >10^5^/μL; ^9^Malaria transmission intensity defined from estimates of *Plasmodium falciparum* prevalence rate (PfPR) according to enrolment year and location, assuming low transmission for study sites with a PfPR <0·10, moderate transmission if PfPR 0·10 to <0·40, and high transmission if PfPR ≥0·40. *Fisher’s exact test

## Supplementary Table 4. Univariable analysis of parasite positivity on Day 3 in Asia.

Logistic regression models with random intercept models for study-site were fitted. Models are adjusted for artemisinin resistance level.

|  | Asia | | | |
| --- | --- | --- | --- | --- |
|  | N | % | OR (95% CI) | P-value |
| Total MQ dose (mg/kg) | 4304 | 5.9 | 0.99 (0.94 - 1.05) | 0.776 |
| Number of MQ doses |  |  |  |  |
| Three | 1538 | 13.3 | 3.24 (1.96 - 5.35) | <0.001 |
| Two | 2766 | 1.7 | Reference |  |
| MQ dose (mg/kg) at 24h | 4304 | 5.9 | 0.89 (0.83 - 0.95) | <0.001 |
| Total MQ dose (mg/kg) over first 24h | 4304 | 5.9 | 1.04 (0.96 - 1.12) | 0.361 |
| AS total dose (mg/kg) | 4304 | 5.9 | 0.98 (0.84 - 1.13) | 0.743 |
| Drug formulation |  |  |  |  |
| Fixed, 3 MQ doses | 414 | 13.8 | 0.53 (0.21 - 1.36) ^1^ | 0.188 |
| Loose, 2 MQ doses | 2766 | 1.7 | 0.23 (0.12 - 0.45) | <0.001 |
| Loose, 3 MQ doses | 1124 | 13.2 | Reference |  |
| Co-blister, 3 MQ doses | 0 |  |  |  |
| Supervision |  |  |  |  |
| Partial | 600 | 5.8 | 2.14 (0.50 - 9.13) | 0.302 |
| Full | 3704 | 5.9 | Reference |  |
| Age (years) | 4304 | 5.9 | 1.00 (0.99 - 1.01) | 0.796 |
| Age group |  |  |  |  |
| < 1 year | 0 |  |  |  |
| 1 to <5 years | 205 | 2.4 | 1.11 (0.42 - 2.92) | 0.839 |
| 5 to <12 years | 619 | 4.8 | 1.47 (0.93 - 2.32) | 0.095 |
| ≥ 12 years | 3479 | 6.2 | Reference |  |
| Underweight (<5 years of age)^2^ |  |  |  |  |
| Yes | 70 | 1.4 | 0.42 (0.04 -5.07) | 0.498 |
| No | 135 | 3.0 | Reference |  |
| Sex |  |  |  |  |
| Male | 2828 | 6.7 | 1.13 (0.79 - 1.61) | 0.512 |
| Female | 1476 | 4.2 | Reference |  |
| Haemoglobin^3^ | 3961 | 5.8 | 1.09 (1.02 - 1.17) | 0.014 |
| Moderate/Severe anaemia ^4^ |  |  |  |  |
| Yes | 490 | 1.6 | 0.47 (0.22 - 1.00) | 0.051 |
| No | 3471 | 6.3 | Reference |  |
| Severe anaemia^5^ |  |  |  |  |
| Yes | 23 | 0 |  | 0.640* |
| No | 3938 | 5.8 |  |  |
| Temperature (C ) | 3634 | 5.9 | 1.52 (1.29 - 1.77) | <0.001 |
| Fever or history of fever^6^ |  |  |  |  |
| Yes | 2215 | 7.0 | 2.27 (1.55 - 3.33) | <0.001 |
| No | 1449 | 4.1 | Reference |  |
| Log10 Parasitaemia (/µL) | 4304 | 5.9 | 3.32 (2.58 - 4.26) | <0.001 |
| Hyperparasitaemia ^7^ |  |  |  |  |
| Yes | 334 | 12.9 | 2.78 (1.81 - 4.27) | <0.001 |
| No | 3970 | 5.3 | Reference |  |
| Gametocytes on enrolment |  |  |  |  |
| Yes | 274 | 6.9 | 0.91 (0.52 - 1.59) | 0.748 |
| No | 3591 | 6.2 | Reference |  |

^1^comparison between Fixed, 3 MQ doses and Loose, 2 MQ doses: OR (95% CI)=2.29 (1.14-4.63), p=0.020; ^2^ Underweight defined in children <5 years of age, as weight-for age z-score <–2; ^3^Ht converted to Hb using the formula $Hematocrit =5\cdot62+2\cdot60 \times Haemoglobin$ (Lee, 2008); ^4^Severe anaemia defined as haemoglobin <7 g/dL or haematocrit<20%; ^5^Moderate anaemia as 7 g/dL ≤haemoglobin <10 g/dL or 20%≤haematocrit<30%; ^6^Defined as temperature >37·5°C or reported history of fever; ^7^Defined as parasitaemia >10^5^/μL *Fisher’s exact test

# Supplementary Table 5. Kaplan Meier estimates of the proportion of patients with recrudescence by day 42.

|  | N studies | N  obs | Naïve KM estimate (95% CI) | Pooled KM estimate (95%CI) | N studies^1^ | N  obs | I^2^ |
| --- | --- | --- | --- | --- | --- | --- | --- |
| By region/ART resistance level | | | | | | | |
| Asia, No ARTr | 14 | 2504 | 2.4 (1.8 – 3.1) | 3.2 (2.1-4.8) | 8 | 2065 | 57% |
| Asia, Low ARTr | 8 | 1968 | 3.5 (2.7 – 4.5) | 3.5 (1.7-7.0) | 5 | 1680 | 83% |
| Asia, High ARTr | 4 | 490 | 16.1 (12.9-19.9) | 5.3 (0.7-37.0) | 3 | 418 | 85% |
| Africa | 8 | 1257 | 2.5 (1.5-4.2) | 2.4 (1.3-4.4) | 2 | 518 | 0% |
| By age group (Asia + Africa, High ARTr excluded) | | | | | | | |
| <1 year | 3 | 61 | 0.0 |  |  |  |  |
| 1-4 years | 20 | 818 | 3.6 (2.4 – 5.3) | 8.5 (3.3-21.2) | 6 | 524 | 75% |
| 5-11 years | 20 | 1115 | 3.6 (2.6 – 5.0) | 6.2 (3.8-10.0) | 10 | 628 | 47% |
| 12+ years | 24 | 3735 | 2.2 (1.8 – 2.8) | 2.3 (1.5 – 3.6) | 11 | 2966 | 61% |
| By age group (Asia) |  |  |  |  |  |  |  |
| <1 year |  |  |  |  |  |  |  |
| 1-4 years | 16 | 280 | 4.7 (2.7-8.1) | 11.9 (4.7-28.1) | 5 | 143 | 57% |
| 5-11 years | 17 | 830 | 5.1 (3.7-6.8) | 6.9 (4.0-11.6) | 9 | 605 | 60% |
| 12+ years | 21 | 3851 | 3.9 (3.3 – 4.6) | 2.4 (1.4-4.1) | 12 | 3298 | 80% |
| By age group (Asia No ARTr) |  |  |  |  |  |  |  |
| <1 year | 1 | 1 | 0.0 |  |  |  |  |
| 1-4 years | 12 | 192 | 2.9 (1.20-6.7) | 7.9 (2.3 -25.1) | 4 | 107 | 49% |
| 5-11 years | 12 | 577 | 4.5 (3.1-6.6) | 6.6 (3.8-11.5) | 8 | 463 | 50% |
| 12+ years | 14 | 1734 | 1.6 (1.1 – 2.4) | 2.2 (1.3-3.5) | 8 | 1472 | 29% |
| By age group (Asia, high ARTr excluded) | | | | | | | |
| <1 year | 1 | 1 | 0.0 |  |  |  |  |
| 1-4 years | 15 | 273 | 4.8 (2.7-8.3) | 12.0 (4.8-28.5) | 5 | 142 | 57% |
| 5-11 years | 16 | 784 | 4.4 (3.2-6.2) | 6.7 (4.1-10.7) | 9 | 575 | 47% |
| 12+ years | 19 | 3414 | 2.3 (1.9-3.0) | 2.3 (1.5-3.6) | 11 | 2933 | 61% |
| By age group ( Africa ) |  |  |  |  |  |  |  |
| <1 year | 2 | 60 | 0.0 |  |  |  |  |
| 1-4 years | 5 | 545 | 3.3 (1.8-5.8) | 2.9 (1.5-5.6) | 1 | 382 |  |
| 5-11 years | 5 | 331 | 3.2 (0.8 – 12.7) | 2.3 (0.3 – 15.1) | 1 | 45 |  |
| 12+ years | 6 | 321 | 0.3 (0.05 -2.4) |  | 0 |  |  |
| By MQ dose in children 1-5 years, 3-dose regimen (Asia+Africa, High ARTr resistance region excluded) | | | | | | | |
| <20 mg/kg | 4 | 99 | 4.3 (1.6 – 11.1) | 33.3 (9.6 – 80.5) | 1 | 6 |  |
| 20 - <25 mg/kg | 8 | 169 | 3.9 (1.6 – 9.4) | 9.1 (0.4-88.3) | 2 | 141 | 90% |
| 25+ mg/kg | 9 | 328 | 3.7 (1.8 – 7.4) | 13.7 (1.0 – 88.8) | 2 | 205 | 85% |

^1^ only studies with recorded recrudescences in the group of interest are included

ARTr = artemisinin resistance level

Naïve KM – Kaplan-Meier estimate from all data combined, unadjusted for studies

Pooled KM – pooled Kaplan Meier estimate from the random effect meta-analysis of study level KM estimates. Studies with no recrudescences recorded, and studies with follow-up <42 days were excluded for computational limitations.

I^2^ – proportion of variability in a meta-analysis that is explained by differences between the included trials

## Supplementary Table 6. Univariable analysis of recrudescence by day 42.

Cox regression models with shared frailty for study-site were fitted. Models for Asia are adjusted for artemisinin resistance level.

|  | Asia | | | | Africa | | | |
| --- | --- | --- | --- | --- | --- | --- | --- | --- |
|  | N | % | HR (95% CI) | P-value | N | % | HR (95% CI) | P-value |
| Total MQ dose (mg/kg) | 4962 | 3.7 | 0.94 (0.88 - 1.00) | 0.061 | 1257 | 1.4 | 1.04 (0.97 - 1.12) | 0.313 |
| Number of MQ doses |  |  |  |  |  |  |  |  |
| Three | 1794 | 6.4 | 1.12 (0.68 - 1.84) | 0.67 | 1257 | 1.4 | Reference |  |
| Two | 3168 | 2.2 | Reference |  |  |  |  |  |
| AS total dose (mg/kg) | 4962 | 3.7 | 1.08 (0.97 - 1.21) | 0.168 | 1257 | 1.4 | 1.17 (1.04 - 1.31) | 0.007 |
| Drug formulation |  |  |  |  |  |  |  |  |
| Fixed, 3 MQ doses | 582 | 1.5 | 0.27 (0.10 - 0.69) ^1^ | 0.007 | 801 | 1.1 | 0.47 (0.15 – 1.45) | 0.188 |
| Loose, 2 MQ doses | 3168 | 2.2 | 0.54 (0.30 - 1.00) | 0.049 |  |  |  |  |
| Loose, 3 MQ doses | 1212 | 8.7 | Reference |  | 78 | 1.3 | Reference^2^ |  |
| Co-blister, 3 MQ doses |  |  |  |  | 378 | 1.9 | Reference^2^ |  |
| Supervision |  |  |  |  |  |  |  |  |
| Partial | 942 | 3.1 | 1.70 (0.62 - 4.68) | 0.304 | 0 |  |  |  |
| Full | 4020 | 3.9 | Reference |  | 1257 | 1.4 |  |  |
| Age (years) | 4962 | 3.7 | 0.96 (0.94 - 0.97) | <0.001 | 1257 | 1.4 | 0.94 (0.85 - 1.04) | 0.241 |
| Age group |  |  |  |  |  |  |  |  |
| < 1 year | 1 | 0.0 |  |  | 60 | 0.0 |  |  |
| 1 to <5 years | 280 | 4.3 | 3.01 (1.60 - 5.64) | <0.001 | 545 | 2.2 | 5.18 (0.61 - 44.28) | 0.133 |
| 5 to <12 years | 830 | 4.8 | 2.20 (1.52 - 3.19) | <0.001 | 331 | 1.2 | 3.91 (0.41 - 37.03) | 0.235 |
| ≥ 12 years | 3851 | 3.5 | Reference |  | 321 | 0.3 | Reference |  |
| Underweight (<5 years of age)^3^ |  |  |  |  |  |  |  |  |
| Yes | 118 | 2.5 | 0.75 (0.18 – 3.13) | 0.692 | 507 | 2.2 | 0.44 (0.06 – 3.42) | 0.434 |
| No | 162 | 5.6 | Reference |  | 97 | 1.0 | Reference |  |
| Sex |  |  |  |  |  |  |  |  |
| Male | 3240 | 3.7 | 0.89 (0.65 - 1.22) | 0.470 | 634 | 1.3 | 0.94 (0.36 - 2.44) | 0.894 |
| Female | 1722 | 3.8 | Reference |  | 623 | 1.4 | Reference |  |
| Haemoglobin^4^ | 4598 | 3.9 | 1.03 (0.96 - 1.10) | 0.459 | 1074 | 1.5 | 1.10 (0.84 - 1.44) | 0.497 |
| Moderate/Severe anaemia ^5^ |  |  |  |  |  |  |  |  |
| Yes | 732 | 2.5 | 1.05 (0.63 - 1.75) | 0.847 | 432 | 0.9 | 0.42 (0.13 - 1.33) | 0.139 |
| No | 3866 | 4.2 | Reference |  | 642 | 1.9 | Reference |  |
| Severe anaemia^6^ |  |  |  |  |  |  |  |  |
| Yes | 37 | 0.0 |  |  | 45 | 2.2 | 1.43 (0.19 - 10.97) | 0.733 |
| No | 4561 | 3.9 |  |  | 1029 | 1.5 | Reference |  |
| Temperature (C ) | 4106 | 4.1 | 1.34 (1.16 - 1.54) | <0.001 | 1224 | 1.4 | 1.93 (1.12 - 3.33) | 0.018 |
| Fever or history of fever^7^ |  |  |  |  |  |  |  |  |
| Yes | 2461 | 4.3 | 1.39 (0.99 - 1.95) | 0.056 | 962 | 1.7 | 3.38 (0.44 - 26.04) | 0.242 |
| No | 1675 | 3.6 | Reference |  | 262 | 0.4 | Reference |  |
| Log10 Parasitaemia (/µL) | 4962 | 3.7 | 1.73 (1.40 - 2.13) | <0.001 | 1257 | 1.4 | 2.18 (0.81 - 5.86) | 0.124 |
| Hyperparasitaemia ^8^ |  |  |  |  |  |  |  |  |
| Yes | 351 | 8.0 | 2.13 (1.42 - 3.20) | <0.001 | 154 | 1.9 | 1.18 (0.32 - 4.33) | 0.807 |
| No | 4611 | 3.4 | Reference |  | 1103 | 1.3 | Reference |  |
| Gametocytes on enrolment |  |  |  |  |  |  |  |  |
| Yes | 452 | 2.0 | 0.48 (0.25 - 0.95) | 0.035 | 60 | 3.3 | 2.35 (0.51 - 10.75) | 0.270 |
| No | 4075 | 4.1 | Reference |  | 1175 | 1.3 | Reference |  |
| Transmission Intensity^9^ |  |  |  |  |  |  |  |  |
| High |  |  |  |  | 256 | 3.5 | 8.21 (2.22 - 30.36) | 0.002 |
| Moderate |  |  |  |  | 322 | 1.6 | 2.01 (0.47 - 8.62) | 0.349 |
| Low | 4962 | 3.7 |  |  | 679 | 0.4 | Reference |  |

^1^comparison between Fixed, 3 MQ doses and Loose, 2 MQ doses: p =0.087 ^2^Coblister and Loose 3 MQ doses groups combined as model not estimable when analysed separately; ^3^Underweight defined in children <5 years of age, as weight-for age z-score <–2;^4^Ht converted to Hb using the formula $Hematocrit =5\cdot62+2\cdot60 \times Haemoglobin$ (Lee, 2008); ^5^Severe anaemia defined as haemoglobin <7 g/dL or haematocrit<20%; ^6^Moderate anaemia as 7 g/dL ≤haemoglobin <10 g/dL or 20%≤haematocrit<30%; ^7^Defined as temperature >37·5°C or reported history of fever; ^8^Defined as parasitaemia >10^5^/μL; ^9^Malaria transmission intensity defined from estimates of *Plasmodium falciparum* prevalence rate (PfPR) according to enrolment year and location, assuming low transmission for study sites with a PfPR <0·10, moderate transmission if PfPR 0·10 to <0·40, and high transmission if PfPR ≥0·40.

## Supplementary Table 7. Univariable analysis of recrudescence by day 28.

Cox regression models with shared frailty for study-site were fitted. Models for Asia are adjusted for artemisinin resistance level.

|  | Asia | | | | Africa | | | |
| --- | --- | --- | --- | --- | --- | --- | --- | --- |
|  | N | % | HR (95% CI) | P-value | N | % | HR (95% CI) | P-value |
| Total MQ dose (mg/kg) | 4976 | 3.0 | 0.94 (0.87 - 1.01) | 0.104 | 1270 | 0.9 | 1.03 (0.93 - 1.14) | 0.555 |
| Number of MQ doses |  |  |  |  |  |  |  |  |
| Three | 1797 | 5.1 | 1.02 (0.57 - 1.81) | 0.952 | 1270 | 0.9 |  |  |
| Two | 3179 | 1.9 | Reference |  | 0 |  |  |  |
| Total AS dose (mg/kg) | 4976 | 3.0 | 1.10 (0.99 - 1.24) | 0.084 | 1270 | 0.9 | 1.17 (1.02 - 1.36) | 0.030 |
| Drug formulation |  |  |  |  |  |  |  |  |
| Fixed, 3 MQ doses | 584 | 0.9 | 0.19 (0.06 - 0.62) ^1^ | 0.006 | 814 | 0.5 | 0.31 (0.08 - 1.17)^2^ | 0.083 |
| Loose, 2 MQ doses | 3179 | 1.9 | 0.58 (0.30 - 1.13) | 0.109 | 0 |  |  |  |
| Loose, 3 MQ doses | 1213 | 7.1 | Reference |  | 78 | 0.0 |  | 0.609* |
| Co-blister, 3 MQ doses |  |  |  |  | 378 | 1.9 | Reference |  |
| Supervision |  |  |  |  |  |  |  |  |
| Partial | 947 | 2.1 | 1.49 (0.49 - 4.51) | 0.480 | 0 |  |  |  |
| Full | 4029 | 3.2 | Reference |  | 1270 | 0.9 |  |  |
| Age (years) | 4976 | 3.0 | 0.95 (0.93 - 0.96) | <0.001 | 1270 | 0.9 | 0.95 (0.85 - 1.06) | 0.351 |
| Age group |  |  |  |  |  |  |  |  |
| < 1 year | 1 | 0.0 |  |  | 60 | 0.0 |  | 1.000* |
| 1 to <5 years | 282 | 3.9 | 3.94 (2.02 - 7.71) | <0.001 | 558 | 1.3 | 4.53 (0.47 - 44.12) | 0.193 |
| 5 to <12 years | 832 | 4.4 | 2.71 (1.83 - 4.03) | <0.001 | 331 | 0.9 | 2.67 (0.24 - 29.83) | 0.425 |
| ≥ 12 years | 3861 | 2.6 | Reference |  | 321 | 0.3 | Reference |  |
| Underweight (<5 years of age)^3^ |  |  |  |  |  |  |  |  |
| Yes | 119 | 2.5 | 0.78 (0.19 – 3.30) | 0.741 | 99 | 0 |  | 0.605* |
| No | 163 | 4.9 | Reference |  | 518 | 1.4 |  |  |
| Sex |  |  |  |  |  |  |  |  |
| Male | 3249 | 3.0 | 0.89 (0.63 - 1.28) | 0.539 | 642 | 0.9 | 1.25 (0.38 - 4.10) | 0.717 |
| Female | 1727 | 3.0 | Reference |  | 628 | 0.8 | Reference |  |
| Haemoglobin^4^ | 4612 | 3.2 | 1.02 (0.95 - 1.10) | 0.506 | 1087 | 1.0 | 1.24 (0.91 - 1.68) | 0.167 |
| Moderate/Severe anaemia ^5^ |  |  |  |  |  |  |  |  |
| Yes | 737 | 2.2 | 1.17 (0.68 - 2.03) | 0.565 | 440 | 0.0 |  | 0.004* |
| No | 3875 | 3.4 | Reference |  | 647 | 1.7 |  |  |
| Severe anaemia^6^ |  |  |  |  |  |  |  |  |
| Yes | 38 | 0.0 |  | 0.633* | 46 | 0.0 |  | 1.000* |
| No | 4574 | 3.2 |  |  | 1041 | 1.1 |  |  |
| Temperature (C ) | 4119 | 3.4 | 1.31 (1.12 - 1.53) | <0.001 | 1237 | 0.9 | 1.80 (0.90 - 3.56) | 0.095 |
| Fever or history of fever^7^ |  |  |  |  |  |  |  |  |
| Yes | 2468 | 3.4 | 1.35 (0.94 - 1.95) | 0.106 | 974 | 1.1 |  | 0.134* |
| No | 1681 | 3.2 | Reference |  | 263 | 0.0 |  |  |
| Log10 Parasitaemia (/µL) | 4976 | 3.0 | 1.98 (1.56 - 2.53) | <0.001 | 1270 | 0.9 | 2.54 (0.71 - 9.06) | 0.152 |
| Hyperparasitaemia ^8^ |  |  |  |  |  |  |  |  |
| Yes | 351 | 7.4 | 2.47 (1.61 - 3.80) | <0.001 | 160 | 1.9 | 3.19 (0.76 - 13.49) | 0.115 |
| No | 4625 | 2.7 | Reference |  | 1110 | 0.7 | Reference |  |
| Gametocytes on enrolment |  |  |  |  |  |  |  |  |
| Yes | 453 | 1.3 | 0.40 (0.17 - 0.90) | 0.028 | 63 | 0.0 |  | 1.000* |
| No | 4087 | 3.4 | Reference |  | 1185 | 0.9 |  |  |
| Transmission Intensity^9^ |  |  |  |  |  |  |  |  |
| High | 0 |  |  |  | 256 | 2.7 | 18.44 (2.27 - 149.86) | 0.006 |
| Moderate | 0 |  |  |  | 327 | 0.9 | 6.26 (0.65 - 60.14) | 0.112 |
| Low | 4976 | 3.0 |  |  | 687 | 0.1 | Reference |  |

^1^comparison between Fixed, 3 MQ doses and Loose, 2 MQ doses: HR 0.33, 95%CI 0.12-0.96, p=0.041; ^2^ comparison between Fixed, 3 MQ doses and combined Loose, 3 MQ doses +Coblister: p =0.260; ^3^Underweight defined in children <5 years of age, as weight-for age z-score <–2;^4^Ht converted to Hb using the formula $Hematocrit =5\cdot62+2\cdot60 \times Haemoglobin$ (Lee, 2008); ^5^Severe anaemia defined as haemoglobin <7 g/dL or haematocrit<20%; ^6^Moderate anaemia as 7 g/dL ≤haemoglobin <10 g/dL or 20%≤haematocrit<30%; ^7^Defined as temperature >37·5°C or reported history of fever; ^8^Defined as parasitaemia >10^5^/μL; ^9^Malaria transmission intensity defined from estimates of *Plasmodium falciparum* prevalence rate (PfPR) according to enrolment year and location, assuming low transmission for study sites with a PfPR <0·10, moderate transmission if PfPR 0·10 to <0·40, and high transmission if PfPR ≥0·40.

*Fisher’s exact test

## Supplementary Table 8. Univariable analysis of recrudescence by day 63.

Cox regression models with shared frailty for study-site were fitted. Models for Asia are stratified by artemisinin resistance level.

|  | Asia | | | | Africa | | | |
| --- | --- | --- | --- | --- | --- | --- | --- | --- |
|  | N | % | HR (95% CI) | P-value | N | % | HR (95% CI) | P-value |
| Total MQ dose (mg/kg) | 4952 | 3.9 | 0.94 (0.88 - 1.00) | 0.054 | 1251 | 1.8 | 1.01 (0.95 - 1.08) | 0.718 |
| Number of MQ doses |  |  |  |  |  |  |  |  |
| Three | 1791 | 6.4 | 1.05 (0.65 - 1.72) | 0.836 | 1251 | 1.8 |  |  |
| Two | 3161 | 2.5 | Reference |  | 0 |  |  |  |
| Total AS dose (mg/kg) | 4952 | 3.9 | 1.08 (0.97 - 1.20) | 0.160 | 1251 | 1.8 | 1.11 (0.99 - 1.24) | 0.072 |
| Drug formulation |  |  |  |  |  |  |  |  |
| Fixed, 3 MQ doses | 581 | 1.7 | 0.29 (0.12 - 0.74) ^1^ | 0.009 | 795 | 1.9 | 1.62 (0.19-13.88)^2^ | 0.660 |
| Loose, 2 MQ doses | 3161 | 2.5 | 0.59 (0.32 - 1.07) | 0.080 | 0 |  |  |  |
| Loose, 3 MQ doses | 1210 | 8.7 | Reference |  | 78 | 1.3 | Reference |  |
| Co-blister, 3 MQ doses |  |  |  |  | 378 | 1.9 | 7.28 (0.62 – 85.63) | 0.114 |
| Supervision |  |  |  |  |  |  |  |  |
| Partial | 938 | 3.3 | 1.59 (0.60 - 4.21) | 0.355 | 0 |  |  |  |
| Full | 4014 | 4.1 | Reference |  | 1251 | 1.8 |  |  |
| Age (years) | 4952 | 3.9 | 0.96 (0.95 - 0.97) | <0.001 | 1251 | 1.8 | 0.95 (0.86 - 1.04) | 0.245 |
| Age group |  |  |  |  |  |  |  |  |
| < 1 year | 1 | 0.0 |  |  | 59 | 0.0 |  | 1.000* |
| 1 to <5 years | 279 | 4.3 | 2.75 (1.48 - 5.13) | 0.001 | 540 | 3.3 | 5.97 (0.69 - 52.02) | 0.106 |
| 5 to <12 years | 828 | 5.0 | 2.12 (1.48 - 3.05) | <0.001 | 331 | 1.2 | 4.34 (0.45 - 42.19) | 0.206 |
| ≥ 12 years | 3844 | 3.7 | Reference |  | 321 | 0.3 | Reference |  |
| Underweight (<5 years of age)^3^ |  |  |  |  |  |  |  |  |
| Yes | 118 | 2.5 | 0.74 (0.18-3.11) | 0.688 | 95 | 2.1 | 0.62 (0.14 – 2.70) | 0.521 |
| No | 161 | 5.6 | Reference |  | 503 | 3.2 | Reference |  |
| Sex | 3232 | 3.9 | 0.89 (0.65 - 1.22) | 0.464 | 630 | 2.1 | 1.37 (0.59 - 3.14) | 0.464 |
| Male | 1720 | 4.0 | Reference |  | 621 | 1.6 | Reference |  |
| Female |  |  |  |  |  |  |  |  |
| Haemoglobin^4^ | 4589 | 4.1 | 1.02 (0.95 - 1.08) | 0.627 | 1068 | 2.1 | 1.13 (0.88 - 1.44) | 0.330 |
| Moderate/Severe anaemia^5^ |  |  |  |  |  |  |  |  |
| Yes | 730 | 2.6 | 1.05 (0.64 - 1.72) | 0.857 | 427 | 1.4 | 0.40 (0.15 - 1.06) | 0.064 |
| No | 3859 | 4.4 | Reference |  | 641 | 2.5 | Reference |  |
| Severe anaemia^6^ |  |  |  |  |  |  |  |  |
| Yes | 37 | 2.7 | 1.74 (0.23 - 13.09) | 0.589 | 45 | 2.2 | 1.03 (0.14 - 7.77) | 0.977 |
| No | 4552 | 4.1 | Reference |  | 1023 | 2.1 | Reference |  |
| Temperature (C ) | 4096 | 4.3 | 1.37 (1.19 - 1.57) | <0.001 | 1218 | 1.9 | 1.85 (1.15 - 2.96) | 0.011 |
| Fever or history of fever^7^ |  |  |  |  |  |  |  |  |
| Yes | 2451 | 4.7 | 1.45 (1.04 - 2.03) | 0.029 | 958 | 2.2 | 2.00 (0.45 - 9.02) | 0.364 |
| No | 1675 | 3.6 | Reference | . | 260 | 0.8 | Reference |  |
| Log10 Parasitaemia (/µL) | 4952 | 3.9 | 1.72 (1.41 - 2.11) | <0.001 | 1251 | 1.8 | 2.94 (1.14 - 7.56) | 0.026 |
| Hyperparasitaemia^8^ |  |  |  |  |  |  |  |  |
| Yes | 351 | 8.5 | 2.19 (1.48 - 3.25) | <0.001 | 151 | 4.0 | 1.54 (0.57 - 4.16) | 0.394 |
| No | 4601 | 3.6 | Reference |  | 1100 | 1.5 | Reference |  |
| Gametocytes on enrolment |  |  |  |  |  |  |  |  |
| Yes | 451 | 2.2 | 0.53 (0.28 - 1.00) | 0.050 | 60 | 3.3 | 1.71 (0.38 - 7.63) | 0.485 |
| No | 4066 | 4.3 | Reference |  | 1169 | 1.8 | Reference |  |
| Transmission Intensity^9^ |  |  |  |  |  |  |  |  |
| High | 0 |  |  |  | 256 | 4.3 | 6.06 (2.10-17.45) | 0.001 |
| Moderate | 0 |  |  |  | 320 | 2.2 | 1.39 (0.43 – 4.48) | 0.580 |
| Low | 4952 | 3.9 |  |  | 675 | 0.7 | Reference |  |

^1^comparison between Fixed, 3 MQ doses and Loose, 2 MQ doses: p=0.079; ^2^ comparison between Fixed, 3 MQ doses and combined Loose, 3 MQ doses +Coblister: p =0.261; ^3^Underweight defined in children <5 years of age, as weight-for age z-score <–2;^4^Ht converted to Hb using the formula $Hematocrit =5\cdot62+2\cdot60 \times Haemoglobin$ (Lee, 2008); ^5^Severe anaemia defined as haemoglobin <7 g/dL or haematocrit<20%; ^6^Moderate anaemia as 7 g/dL ≤haemoglobin <10 g/dL or 20%≤haematocrit<30%; ^7^Defined as temperature >37·5°C or reported history of fever; ^8^Defined as parasitaemia >10^5^/μL; ^9^Malaria transmission intensity defined from estimates of *Plasmodium falciparum* prevalence rate (PfPR) according to enrolment year and location, assuming low transmission for study sites with a PfPR <0·10, moderate transmission if PfPR 0·10 to <0·40, and high transmission if PfPR ≥0·40.

*Fisher’s exact test

## Supplementary Table 9. Univariable analysis of reinfection by day 42.

Cox regression models with shared frailty for study-site were fitted.

|  | Asia | | | | Africa | | | |
| --- | --- | --- | --- | --- | --- | --- | --- | --- |
|  | N | % | HR (95% CI) | P-value | N | % | HR (95% CI) | P-value |
| Total MQ dose (mg/kg) | 4952 | 4.0 | 0.97 (0.92 - 1.02) | 0.222 | 1251 | 13.2 | 0.99 (0.96 - 1.02) | 0.404 |
| Number of MQ doses |  |  |  |  |  |  |  |  |
| Three | 1791 | 4.2 | 0.85 (0.52 - 1.37) | 0.500 | 1251 | 13.2 |  |  |
| Two | 3161 | 3.8 | Reference |  | 0 |  |  |  |
| Drug formulation |  |  |  |  |  |  |  |  |
| Fixed, 3 MQ doses | 581 | 2.2 | 0.53 (0.22 - 1.26) ^1^ | 0.151 | 795 | 16.6 | 3.85 (0.59-25.14)^2^ | 0.159 |
| Loose, 2 MQ doses | 3161 | 3.8 | 0.93 (0.52 - 1.66) | 0.798 | 0 |  |  |  |
| Loose, 3 MQ doses | 1210 | 5.1 | Reference |  | 78 | 5.1 | Reference |  |
| Co-blister, 3 MQ doses |  |  |  |  | 378 | 7.7 | 2.22 (0.26-19.15) | 0.469 |
| Supervision |  |  |  |  |  |  |  |  |
| Partial | 938 | 3.1 | 0.70 (0.31 - 1.59) | 0.393 | 0 |  |  |  |
| Full | 4014 | 4.2 | Reference |  | 1251 | 13.2 |  |  |
| Age (years) | 4952 | 4.0 | 0.98 (0.96 - 0.99) | <0.001 | 1251 | 13.2 | 0.87 (0.81 - 0.93) | <0.001 |
| Age group |  |  |  |  |  |  |  |  |
| < 1 year | 1 | 0.0 |  |  | 59 | 28.8 | 23.66 (4.80 - 116.50) | <0.001 |
| 1 to <5 years | 279 | 5.0 | 2.02 (1.12 - 3.64) | 0.019 | 540 | 24.3 | 23.15 (5.06 - 105.95) | <0.001 |
| 5 to <12 years | 828 | 4.0 | 1.32 (0.88 - 1.96) | 0.175 | 331 | 4.5 | 6.59 (1.40 - 31.12) | 0.017 |
| ≥ 12 years | 3844 | 3.9 | Reference |  | 321 | 0.6 | Reference |  |
| Underweight (<5 years of age)^3^ |  |  |  |  |  |  |  |  |
| Yes |  |  |  |  |  |  |  |  |
| No |  |  |  |  |  |  |  |  |
| Sex |  |  |  |  |  |  |  |  |
| Male | 3232 | 4.3 | 1.44 (1.04 - 1.99) | 0.029 | 630 | 12.9 | 1.10 (0.81 - 1.50) | 0.536 |
| Female | 1720 | 3.3 | Reference |  | 621 | 13.5 | Reference |  |
| Transmission Intensity^9^ |  |  |  |  |  |  |  |  |
| High | 0 |  |  |  | 256 | 19.9 | 3.47 (0.53 - 22.81) | 0.195 |
| Moderate | 0 |  |  |  | 320 | 23.1 | 1.43 (0.39 - 5.23) | 0.589 |
| Low | 4952 | 4.0 |  |  | 675 | 5.9 | Reference |  |

^1^comparison between Fixed, 3 MQ doses and Loose, 2 MQ doses: p=0.136; ^2^ comparison between Fixed, 3 MQ doses and combined Loose, 3 MQ doses +Coblister: p =0.211; ^3^Underweight defined in children <5 years of age, as weight-for age z-score <–2;^4^Ht converted to Hb using the formula $Hematocrit =5\cdot62+2\cdot60 \times Haemoglobin$ (Lee, 2008); ^5^Severe anaemia defined as haemoglobin <7 g/dL or haematocrit<20%; ^6^Moderate anaemia as 7 g/dL ≤haemoglobin <10 g/dL or 20%≤haematocrit<30%; ^7^Defined as temperature >37·5°C or reported history of fever; ^8^Defined as parasitaemia >10^5^/μL; ^9^Malaria transmission intensity defined from estimates of *Plasmodium falciparum* prevalence rate (PfPR) according to enrolment year and location, assuming low transmission for study sites with a PfPR <0·10, moderate transmission if PfPR 0·10 to <0·40, and high transmission if PfPR ≥0·40.

## Supplementary Table 10. Univariable analysis of reinfection by day 28.

Cox regression models with shared frailty for study-site were fitted.

|  | Asia | | | | | | | Africa | | | | | |
| --- | --- | --- | --- | --- | --- | --- | --- | --- | --- | --- | --- | --- | --- |
|  | N | % | | HR (95% CI) | | P-value | | N | | % | | HR (95% CI) | P-value |
| Total MQ dose (mg/kg) | 4952 | 1.5 | | 0.91 (0.82 - 1.00) | | 0.053 | | 1251 | | 7.9 | | 0.97 (0.94 - 1.01) | 0.121 |
| Number of MQ doses |  |  | |  | |  | |  | |  | |  |  |
| Three | 1791 | 2.2 | | 1.42 (0.68 - 2.95) | | 0.347 | | 1251 | | 7.9 | |  |  |
| Two | 3161 | 1.2 | | Reference | |  | |  | |  | |  |  |
| Drug formulation |  |  | |  | |  | |  | |  | |  |  |
| Fixed, 3 MQ doses | 581 | 1.0 | | 0.65 (0.18 - 2.28) ^1^ | | 0.502 | | 795 | | 8.8 | | 3.06 (0.54 -17.30) ^2^ | 0.207 |
| Loose, 2 MQ doses | 3161 | 1.2 | | 0.61 (0.26 - 1.41) | | 0.246 | |  | |  | |  |  |
| Loose, 3 MQ doses | 1210 | 2.7 | | Reference | |  | | 78 | | 0.0 | | Reference |  |
| Co-blister, 3 MQ doses | 0 |  | |  | |  | | 378 | | 7.7 | | Reference |  |
| Supervision |  |  | |  | |  | |  | |  | |  |  |
| Partial | 938 | 0.9 | | 0.51 (0.15 - 1.75) | | 0.285 | | 0 | |  | |  |  |
| Full | 4014 | 1.7 | | Reference | |  | | 1251 | | 7.9 | |  |  |
| Age (years) | 4952 | 1.5 | | 0.96 (0.94 - 0.98) | | <0.001 | | 1251 | | 7.9 | | 0.85 (0.78 - 0.93) | <0.001 |
| Age group |  |  | |  | |  | |  | |  | |  |  |
| < 1 year | 1 | 0.0 | |  | |  | | 59 | | 15.3 | | 17.32 (3.06 - 98.14) | 0.001 |
| 1 to <5 years | 279 | 1.8 | | 3.06 (1.14 - 8.21) | | 0.026 | | 540 | | 14.3 | | 14.67 (2.97 - 72.56) | <0.001 |
| 5 to <12 years | 828 | 1.9 | | 2.13 (1.16 - 3.89) | | 0.014 | | 331 | | 3.3 | | 3.69 (0.72 - 19.01) | 0.118 |
| ≥ 12 years | 3844 | 1.4 | | Reference | |  | | 321 | | 0.6 | | Reference |  |
| Underweight (<5 years of age)^3^ |  | |  | |  | |  | |  | |  |  |  |
| Yes | 118 | | 0.9 | | 0.37 (0.4 -3.53) | | 0.385 | | 95 | | 12.6 | 0.81 (0.44 – 1.49) | 0.493 |
| No | 161 | | 2.5 | | Reference | |  | | 503 | | 14.7 | Reference |  |
| Sex |  |  | |  | |  | |  | |  | |  |  |
| Male | 3232 | 1.7 | | 1.24 (0.73 - 2.10) | | 0.420 | | 630 | | 7.9 | | 1.19 (0.80 - 1.77) | 0.388 |
| Female | 1720 | 1.3 | | Reference | |  | | 621 | | 7.9 | | Reference |  |
| Transmission Intensity^9^ |  |  | |  | |  | |  | |  | |  |  |
| High | 0 |  | |  | |  | | 256 | | 14.8 | | 3.29 (0.21 - 52.76) | 0.400 |
| Moderate | 0 |  | |  | |  | | 320 | | 13.4 | | 1.50 (0.22 - 10.13) | .675 |
| Low | 4952 | 1.5 | |  | |  | | 675 | | 2.7 | | Reference |  |

^1^comparison between Fixed, 3 MQ doses and Loose, 2 MQ doses: p =0.799; ^2^ comparison between Fixed, 3 MQ doses and Loose, 3 MQ doses: p =0.002*, comparison between Fixed, 3 MQ doses and Coblister: p =0.799; ^3^Underweight defined in children <5 years of age, as weight-for age z-score <–2;^4^Ht converted to Hb using the formula $Hematocrit =5\cdot62+2\cdot60 \times Haemoglobin$ (Lee, 2008); ^5^Severe anaemia defined as haemoglobin <7 g/dL or haematocrit<20%; ^6^Moderate anaemia as 7 g/dL ≤haemoglobin <10 g/dL or 20%≤haematocrit<30%; ^7^Defined as temperature >37·5°C or reported history of fever; ^8^Defined as parasitaemia >10^5^/μL; ^9^Malaria transmission intensity defined from estimates of *Plasmodium falciparum* prevalence rate (PfPR) according to enrolment year and location, assuming low transmission for study sites with a PfPR <0·10, moderate transmission if PfPR 0·10 to <0·40, and high transmission if PfPR ≥0·40.

*Fisher’s exact test

## Supplementary Table 11. Univariable analysis of reinfection by day 63.

Cox regression models with shared frailty for study-site were fitted.

|  | Asia | | | | Africa | | | |
| --- | --- | --- | --- | --- | --- | --- | --- | --- |
|  | N | % | HR (95% CI) | P-value | N | % | HR (95% CI) | P-value |
| Total MQ dose (mg/kg) | 4952 | 6.0 | 0.98 (0.95 - 1.02) | 0.409 | 1251 | 16.5 | 0.99 (0.97 - 1.02) | 0.638 |
| Number of MQ doses |  |  |  |  |  |  |  |  |
| Three | 1791 | 6.5 | 0.90 (0.61 - 1.32) | 0.580 | 1251 | 16.5 | Reference |  |
| Two | 3161 | 5.7 | Reference |  |  |  |  |  |
| Drug formulation |  |  |  |  |  |  |  |  |
| Fixed, 3 MQ doses | 581 | 6.7 | 1.03 (0.53 - 2.01) ^1^ | 0.935 | 795 | 21.9 | 3.00 (0.61 – 26.15)^2^ | 0.149 |
| Loose, 2 MQ doses | 3161 | 5.7 | 1.13 (0.67 - 1.92) | 0.648 | 0 |  |  |  |
| Loose, 3 MQ doses | 1210 | 6.4 | Reference |  | 78 | 5.1 | Reference |  |
| Co-blister, 3 MQ doses |  |  |  |  | 378 | 7.7 | 2.17 (0.25 – 18.82) | 0.481 |
| Supervision |  |  |  |  |  |  |  |  |
| Partial | 938 | 6.4 | 0.79 (0.37 - 1.66) | 0.526 | 0 |  |  |  |
| Full | 4014 | 5.9 | Reference |  | 1251 | 16.5 |  |  |
| Age (years) | 4952 | 6.0 | 0.98 (0.97 - 0.99) | <0.001 | 1251 | 16.5 | 0.88 (0.83 - 0.94) | <0.001 |
| Age group |  |  |  |  |  |  |  |  |
| < 1 year | 1 | 0.0 | 0.00 (0.00 - 0.00) |  | 59 | 35.6 | 23.82 (4.95 - 114.54) | <0.001 |
| 1 to <5 years | 279 | 6.5 | 1.53 (0.91 - 2.55) | 0.105 | 540 | 31.3 | 25.66 (5.66 - 116.25) | <0.001 |
| 5 to <12 years | 828 | 5.6 | 1.26 (0.90 - 1.75) | 0.179 | 331 | 4.5 | 7.12 (1.51 - 33.56) | 0.013 |
| ≥ 12 years | 3844 | 6.0 | Reference |  | 321 | 0.6 | Reference |  |
| Underweight (<5 years of age)^2^ |  |  |  |  |  |  |  |  |
| Yes | 118 | 3.4 | 0.38 (0.12-1.17) | 0.093 | 95 | 36.8 | 1.06 (0.73 -1.53) | 0.761 |
| No | 161 | 8.7 | Reference |  | 503 | 30.8 | Reference |  |
| Sex |  |  |  |  |  |  |  |  |
| Male | 3232 | 6.4 | 1.40 (1.08 - 1.83) | 0.012 | 630 | 16.3 | 1.09 (0.83 - 1.43) | 0.547 |
| Female | 1720 | 5.2 | Reference |  | 621 | 16.7 | Reference |  |
| Transmission Intensity |  |  |  |  |  |  |  |  |
| High | 0 |  |  |  | 256 | 23.8 | 3.13 (0.47 - 21.02) | 0.239 |
| Moderate | 0 |  |  |  | 320 | 26.9 | 1.09 (0.30 - 4.02) | 0.898 |
| Low | 4952 | 6.0 | Reference |  | 675 | 8.9 | Reference |  |

^1^comparison between Fixed, 3 MQ doses and Loose, 2 MQ doses: p =0.702; ^2^comparison between Fixed, 3 MQ doses and combined Loose, 3 MQ doses +Coblister: p =0.181; ^3^Underweight defined in children <5 years of age, as weight-for age z-score <–2;^4^Ht converted to Hb using the formula $Hematocrit =5\cdot62+2\cdot60 \times Haemoglobin$ (Lee, 2008); ^5^Severe anaemia defined as haemoglobin <7 g/dL or haematocrit<20%; ^6^Moderate anaemia as 7 g/dL ≤haemoglobin <10 g/dL or 20%≤haematocrit<30%; ^7^Defined as temperature >37·5°C or reported history of fever; ^8^Defined as parasitaemia >10^5^/μL; ^9^Malaria transmission intensity defined from estimates of *Plasmodium falciparum* prevalence rate (PfPR) according to enrolment year and location, assuming low transmission for study sites with a PfPR <0·10, moderate transmission if PfPR 0·10 to <0·40, and high transmission if PfPR ≥0·40.

## Supplementary Table 12. Univariable analysis of presence of gametocytes on day 7 in patients with gametocytaemia on enrollment.

Logistic regression models with random intercept for study were fitted. **All results adjusted for primaquine administration.**

|  | All regions | | | |
| --- | --- | --- | --- | --- |
|  | N | N | OR(95% CI) | P-value |
| Total MQ dose (mg/kg) | 443 | 23.3 | 1.02 (0.97 - 1.08) | 0.391 |
|  |  |  |  |  |
| Number of MQ doses | 266 | 24.8 | 2.13 (0.86 - 5.27) | 0.101 |
|  | 177 | 20.9 | Reference |  |
| MQ dose at 24h | 443 | 23.3 | 0.96 (0.86 - 1.06) | 0.387 |
| MQ dose over 24h | 443 | 23.3 | 1.05 (0.97 - 1.14) | 0.260 |
| Total AS dose (mg/kg) | 443 | 23.3 | 1.06 (0.96 - 1.18) | 0.263 |
| Primaquine administration |  |  |  |  |
| Yes | 30 | 10.0 | 0.10 (0.02 – 0.41) | 0.002 |
| No | 413 | 24.2 | Reference |  |
| Drug formulation |  |  |  |  |
| Fixed, 3 MQ doses | 177 | 28.2 | 1.64 (0.50-5.34) | 0.413 |
| Loose, 2 MQ doses | 177 | 20.9 | 0.66 (0.20-2.17) | 0.496 |
| Loose, 3 MQ doses | 85 | 17.6 | Reference |  |
| Coblister, 3 MQ doses | 4 | 25.0 | 1.47 (0.10-22.73) | 0.782 |
|  |  |  |  |  |
| Supervision | 113 | 32.7 | 1.81 (0.79 - 4.12) | 0.159 |
|  | 330 | 20.0 | Reference |  |
| Age (years) | 443 | 23.3 | 0.99 (0.98 - 1.01) | 0.466 |
| Age group |  |  |  |  |
| < 1 year | 3 | 0 |  |  |
| 1 to <5 years | 80 | 23.8 | 0.99 (0.47 - 2.08) | 0.973 |
| 5 to <12 years | 107 | 28.0 | 1.20 (0.67 - 2.16) | 0.538 |
| ≥ 12 years | 253 | 21.3 | Reference |  |
| Underweight (<5 years of age)^2^ |  |  |  |  |
| Yes | 33 | 30.3 | 2.54 (0.66 – 9.78) | 0.174 |
| No | 50 | 18.0 | Reference |  |
| Sex |  |  |  |  |
| Male | 263 | 25.5 | 1.58 (0.96 - 2.61) | 0.071 |
| Female | 180 | 20.0 | Reference |  |
| Haemoglobin^3^ | 422 | 23.2 | 1.00 (0.88 - 1.12) | 0.939 |
| Moderate/Severe anaemia ^4^ |  |  |  |  |
| Yes | 179 | 25.7 | 1.08 (0.62 - 1.87) | 0.779 |
| No | 243 | 21.4 | Reference |  |
| Severe anaemia^5^ |  |  |  |  |
| Yes | 17 | 29.4 | 1.20 (0.38 - 3.80) | 0.752 |
| No | 405 | 23.0 | Reference |  |
| Temperature (C ) | 379 | 22.2 | 0.85 (0.66 - 1.09) | 0.204 |
| Fever or history of fever^6^ |  |  |  |  |
| Yes | 242 | 22.3 | 0.98 (0.56 - 1.71) | 0.948 |
| No | 140 | 21.4 | Reference |  |
| Log10 Parasitaemia (/µL) | 443 | 23.3 | 1.02 (0.72 - 1.43) | 0.921 |
| Hyperparasitaemia ^7^ |  |  |  |  |
| Yes | 11 | 27.3 | 2.08 (0.45 - 9.52) | 0.346 |
| No | 432 | 23.1 | Reference |  |
| Transmission Intensity^8^ |  |  |  |  |
| High | 2 | 0 |  |  |
| Moderate | 75 | 16.0 | 0.69 (0.22 - 2.12) | 0.518 |
| Low | 366 | 24.9 | Reference |  |
| Region |  |  |  |  |
| Africa | 51 | 17.7 | Reference |  |
| Asia | 294 | 24.5 | 1.54 (0.41-5.82) | 0.521 |
| South America | 98 | 22.5 | 1.27 (0.23-6.90) | 0.785 |
| Artemisinin resistance level^9^ |  |  |  |  |
| None | 327 | 23.6 | Reference |  |
| Low | 92 | 28.3 | 1.29 (0.50 -3.36) | 0.602 |
| High | 24 | 0 |  | 0.004*^10^ |

*Fisher’s exact test

^1^comparison between Fixed, 3 MQ doses and Loose, 2 MQ doses: p =0.065; ^2^Underweight defined in children <5 years of age, as weight-for age z-score <–2; ^3^Ht converted to Hb using the formula $Hematocrit =5\cdot62+2\cdot60 \times Haemoglobin$ (Lee, 2008); ^4^Severe anaemia defined as haemoglobin <7 g/dL or haematocrit<20%; ^5^Moderate anaemia as 7 g/dL ≤haemoglobin <10 g/dL or 20%≤haematocrit<30%; ^6^Defined as temperature >37·5°C or reported history of fever; ^7^Defined as parasitaemia >10^5^/μL; ^8^Malaria transmission intensity defined from estimates of *Plasmodium falciparum* prevalence rate (PfPR) according to enrolment year and location, assuming low transmission for study sites with a PfPR <0·10, moderate transmission if PfPR 0·10 to <0·40, and high transmission if PfPR ≥0·40. ^9^None: 1995-1999 in Cambodia + 1995-2005 in other sites, Low: 2000-2004 in Cambodia + 2005-2010 in other sites, High: 2005-2018 in Cambodia + 2011-2018 in other sites; ^10^p=0.017* in those who have not received primaquine (n=345).

## Supplementary Table 13. Univariable analysis of presence of gametocytes on days 7-14 in patients without gametocytaemia on enrollment.

Logistic (OR) and Poisson (RR) regression models with random intercept for study-site were fitted. Studies with primaquine administration (n=2) were excluded as no gametocytes carriage on day 7-14 was observed.

|  | All regions (N=3,228, n=28, 21 studies) ^1^ | | | | | | | All regions (N=4232, n=39, 21 studies) | | | | | |
| --- | --- | --- | --- | --- | --- | --- | --- | --- | --- | --- | --- | --- | --- |
|  | N | % | | OR (95% CI) | | P-value | | N | | % | | IRR (95% CI) | P-value |
| Total MQ dose (mg/kg) | 3218 | 0.9 | | 0.96 (0.88 - 1.06) | | 0.457 | | 4232 | | 0.9 | | 0.97 (0.89 - 1.06) | 0.476 |
| Number of MQ doses |  |  | |  | |  | |  | |  | |  |  |
| Three | 1970 | 0.9 | | 0.48 (0.13 - 1.78) | | 0.275 | | 2320 | | 0.8 | | 0.55 (0.22 - 1.41) | 0.215 |
| Two | 1248 | 1.0 | | Reference | |  | | 1912 | | 1.1 | | Reference |  |
| MQ dose (mg/kg) at 24h | 3218 | 0.9 | | 1.03 (0.89 - 1.19) | | 0.705 | | 4232 | | 0.9 | | 1.04 (0.93 - 1.16) | 0.518 |
| Total MQ dose (mg/kg) over first 24h | 3218 | 0.9 | | 0.93 (0.81 - 1.08) | | 0.366 | | 4232 | | 0.9 | | 0.94 (0.82 - 1.07) | 0.349 |
| Total AS dose (mg/kg) | 3218 | 0.9 | | 0.92 (0.76 - 1.10) | | 0.345 | | 4232 | | 0.9 | | 0.92 (0.78 - 1.09) | 0.338 |
| Drug formulation^2^ |  |  | |  | |  | |  | |  | |  |  |
| Fixed, 3 MQ doses | 984 | 0.9 | | 0.21 (0.02 - 1.92) | | 0.168 | | 1142 | | 0.8 | | 0.28 (0.04 - 1.88) | 0.189 |
| Loose, 2 MQ doses | 1248 | 1.0 | | 0.46 (0.05 - 4.19) | | 0.488 | | 1912 | | 1.1 | | 0.53 (0.08 - 3.39) | 0.499 |
| Loose, 3 MQ doses | 625 | 0.3 | | 0.10 (0.01 - 1.73) | | 0.112 | | 810 | | 0.4 | | 0.15 (0.02 - 1.47) | 0.104 |
| Co-blister, 3 MQ doses | 361 | 1.7 | | Reference | |  | | 368 | | 1.6 | | Reference |  |
| Underweight (<5 years of age)^3^ |  | |  | |  | |  | |  | |  |  |  |
| Yes | 102 | | 2.0 | | 6.04 (0.64 -56.73) | | 0.116 | | 137 | | 2.2 | 6.06 (0.97 -37.99) | 0.054 |
| No | 452 | | 0.2 | | Reference | |  | | 529 | | 0.4 |  |  |
| Supervision |  |  | |  | |  | |  | |  | |  |  |
| Partial | 461 | 1.5 | | 3.43 (0.56 - 20.80) | | 0.181 | | 745 | | 1.9 | | 2.82 (1.06 - 7.50) | 0.038 |
| Full | 2757 | 0.8 | | Reference | |  | | 3487 | | 0.7 | | Reference |  |
| Age (years) | 3218 | 0.9 | | 0.99 (0.96 - 1.02) | | 0.581 | | 4232 | | 0.9 | | 0.99 (0.97 - 1.02) | 0.511 |
| Age group |  |  | |  | |  | |  | |  | |  |  |
| < 1 year | 0 |  | |  | |  | | 44 | | 0.0 | |  |  |
| 1 to <5 years | 518 | 0.6 | | 0.59 (0.12 - 2.94) | | 0.520 | | 624 | | 0.8 | | 0.92 (0.30 - 2.83) | 0.888 |
| 5 to <12 years | 644 | 1.6 | | 1.86 (0.72 - 4.78) | | 0.200 | | 850 | | 1.3 | | 1.28 (0.59 - 2.79) | 0.531 |
| ≥ 12 years | 2018 | 0.8 | | Reference | |  | | 2714 | | 0.9 | | Reference |  |
| Sex |  |  | |  | |  | |  | |  | |  |  |
| Male | 1855 | 0.9 | | 1.09 (0.49 - 2.41) | | 0.833 | | 2487 | | 0.8 | | 0.96 (0.50 - 1.84) | 0.897 |
| Female | 1363 | 1.0 | | Reference | |  | | 1745 | | 1.0 | | Reference |  |
| Haemoglobin^4^ | 2916 | 1.0 | | 0.80 (0.67 - 0.97) | | 0.023 | | 3890 | | 0.9 | | 0.81 (0.70 - 0.95) | 0.008 |
| Moderate/Severe anaemia^5^ |  |  | |  | |  | |  | |  | |  |  |
| Yes | 507 | 1.0 | | 1.08 (0.36 - 3.21) | | 0.892 | | 708 | | 1.1 | | 1.11 (0.48 - 2.56) | 0.805 |
| No | 2409 | 1.0 | | Reference | |  | | 3182 | | 0.9 | | Reference |  |
| Severe anaemia^6^ |  |  | |  | |  | |  | |  | |  |  |
| Yes | 37 | 2.7 | | 2.36 (0.15 - 36.86) | | 0.540 | | 44 | | 2.3 | | 2.79 (0.36 - 21.38) | 0.324 |
| No | 2879 | 0.9 | | Reference | |  | | 3846 | | 1.0 | | Reference |  |
| Temperature (C ) | 3127 | 0.9 | | 0.73 (0.47 - 1.15) | | 0.173 | | 4105 | | 0.9 | | 0.84 (0.61 - 1.15) | 0.276 |
| Fever or history of fever^7^ |  |  | |  | |  | |  | |  | |  |  |
| Yes | 2045 | 0.9 | | 0.68 (0.26 - 1.80) | | 0.440 | | 2625 | | 0.9 | | 0.78 (0.37 - 1.67) | 0.528 |
| No | 1082 | 0.8 | | Reference | |  | | 1480 | | 0.9 | | Reference |  |
| Log10 Parasitaemia (/µL) | 3218 | 0.9 | | 0.91 (0.55 - 1.52) | | 0.720 | | 4232 | | 0.9 | | 0.88 (0.58 - 1.33) | 0.532 |
| Hyperparasitaemia ^8^ |  |  | |  | |  | |  | |  | |  |  |
| Yes | 291 | 0.3 | | 0.42 (0.05 - 3.27) | | 0.407 | | 346 | | 0.3 | | 0.35 (0.05 - 2.65) | 0.312 |
| No | 2927 | 1.0 | | Reference | |  | | 3868 | | 1.0 | | Reference |  |
| Transmission Intensity^9^ |  |  | |  | |  | |  | |  | |  |  |
| High | 224 | 1.8 | | 2.28 (0.08 - 63.51) | | 0.627 | | 244 | | 1.6 | | 1.41 (0.16 - 12.18) | 0.752 |
| Moderate | 397 | 1.0 | | 2.36 (0.21 - 26.04) | | 0.483 | | 421 | | 1.2 | | 2.01 (0.34 - 11.83) | 0.442 |
| Low | 2597 | 0.8 | | Reference | |  | | 3567 | | 0.8 | | Reference |  |
| Region |  |  | |  | |  | |  | |  | |  |  |
| Africa | 995 | 0.7 | | Reference | |  | | 1061 | | 0.7 | | Reference |  |
| Asia | 1912 | 0.8 | | 2.26 (0.35-14.74) | | 0.395 | | 2853 | | 0.9 | | 1.52 (0.45 – 5.07) | 0.498 |
| South America | 311 | 1.9 | | 7.39 (0.28-196.67) | | 0.232 | | 318 | | 2.2 | | 4.15 (0.53 – 32.42) | 0.175 |
| Artemisinin resistance level^10^ |  |  | |  | |  | |  | |  | |  |  |
| None | 2626 | 1.3 | | Reference | |  | | 3286 | | 1.0 | | Reference |  |
| Low | 460 | 1.1 | | 0.88 (0.22 – 3.53) | | 0.860 | | 736 | | 0.7 | | 0.75 (0.24 – 2.34) | 0.618 |
| High | 143 | 0 | |  | | 0.418* | | 210 | | 0 | |  |  |

^1^only patients with measurements on days 7 and 14 were included ^2^comparison between Fixed, 3 MQ doses and Loose, 2 MQ doses: p =0.311 in the logistic regression, and p =0.324 in the Poisson regression; ^3^Underweight defined in children <5 years of age, as weight-for age z-score <–2;^4^Ht converted to Hb using the formula $Hematocrit =5\cdot62+2\cdot60 \times Haemoglobin$ (Lee, 2008); ^5^Severe anaemia defined as haemoglobin <7 g/dL or haematocrit<20%; ^6^Moderate anaemia as 7 g/dL ≤haemoglobin <10 g/dL or 20%≤haematocrit<30%; ^7^Defined as temperature >37·5°C or reported history of fever; ^8^Defined as parasitaemia >10^5^/μL; ^9^Malaria transmission intensity defined from estimates of *Plasmodium falciparum* prevalence rate (PfPR) according to enrolment year and location, assuming low transmission for study sites with a PfPR <0·10, moderate transmission if PfPR 0·10 to <0·40, and high transmission if PfPR ≥0·40. ^10^ None: 1995-1999 in Cambodia + 1995-2005 in other sites, Low: 2000-2004 in Cambodia + 2005-2010 in other sites, High: 2005-2018 in Cambodia + 2011-2018 in other sites

*Fisher’s exact test

## Supplementary Table 14. Multivariable analysis for Gametocyte carriage

| Parameter |  | Gametocyte Positivity on Day 7*  (N=441, n=103, 18 studies) | | | Development of Gametocytes**  (N=3890, n=39, 21 studies) | | |
| --- | --- | --- | --- | --- | --- | --- | --- |
|  |  | AOR | 95% CI | P-value | ARR | 95%CI | P-value |
| Total AS dose (mg/kg) |  | 1.04 | 0.86-1.26 | 0.694 | 1.02 | 0.81-1.30 | 0.850 |
| Total MQ dose (mg/kg) |  | 1.01^3^ | 0.91-1.12 | 0.839 | 0.93^4^ | 0.83 – 1.06 | 0.291 |
| Haemoglobin on Day 0 |  |  |  |  | 0.75 | 0.64 – 0.88 | <0.001 |
| Primaquine (single low dose) |  | 0.11 | 0.03-0.46 | 0.002 | NA |  |  |
| Drug formulation |  |  |  |  |  |  |  |
| Fixed, 3 MQ doses |  | 2.86 | 1.12-7.31 | 0.028 |  |  |  |
| Loose, 2 MQ doses |  | 0.43 | 0.16-1.15 | 0.093 |  |  |  |
| Loose, 3 MQ doses^1^ |  | Reference |  |  |  |  |  |
| Region |  |  |  |  |  |  |  |
| Africa |  | Reference |  |  | Reference |  |  |
| South America |  | 1.95 | 0.72-7.50 | 0.275 | 7.08 | 1.15-43.74 | 0.035 |
| Asia, No artemisinin resistance |  | 7.50 | 2.30-35.56 | 0.004 | 2.38 | 0.70 – 8.16 | 0.167 |
| Asia, artemisinin resistance^2^ |  | 2.80 | 1.09 – 10.68 | 0.079 | 1.88 | 0.44 – 8.09 | 0.399 |

N=number of patients included, n=number of events observed

^1^includes coblister formulation

^2^includes regions with low and high resistance due to computational limitations. All 24 patients in high artemisinin resistance areas cleared gametocytes by day 7).

^3^ all other representation of MQ dosing showed similar nonsignificant results and the same AIC

^4^ all other representation of MQ dosing showed similar results nonsignificant and AIC in range 421-422.

*Logistic regression model with random intercept for study was fitted for gametocyte positivity on Day 7 in patients who were gametocytaemic on enrolment, i.e. measuring persistence of gametocyte carriage. Model was adjusted for SLD primaquine use.

**Development of gametocytes after treatment, as measured by carriage of gametocytes (ARR) on days 7 or 14 in patients without gametocytes on enrolment, was modelled using Poisson regression with random intercept for study site. Patients who received primaquine were excluded, as no gametocytes on days 7 or 14 were detected.

## Supplementary Table 15. Univariable analysis of presence of anaemia on day 7.

Logistic regression models with random intercept for study-site were fitted. Models adjusted for baseline anaemia.

|  | All regions | | | |
| --- | --- | --- | --- | --- |
|  | N | n | OR(95% CI) | P-value |
| Total MQ dose (mg/kg) | 3798 | 25.7 | 1.06 (1.04 - 1.09) | <0.001 |
| Number of MQ doses |  |  |  |  |
| Three | 2138 | 30.5 | 1.13 (0.81 - 1.58) | 0.473 |
| Two | 1660 | 19.5 | Reference |  |
| MQ dose (mg/kg) at 24h | 3798 | 25.7 | 1.04 (1.01 - 1.08) | 0.025 |
| Total MQ dose (mg/kg) over first 24h | 3798 | 25.7 | 1.09 (1.05 - 1.13) | <0.001 |
| Total AS dose (mg/kg) | 3798 | 25.7 | 1.17 (1.11 - 1.23) | <0.001 |
| Primaquine |  |  |  |  |
| Yes | 71 | 8.5 | 0.38 (0.08 – 1.74) | 0.213 |
| No | 3727 | 26.0 | Reference |  |
| Drug formulation |  |  |  |  |
| Fixed, 3 MQ doses | 922 | 50.4 | 2.07 (1.25 – 3.43) ^1^ | 0.005 |
| Loose, 2 MQ doses | 1660 | 19.5 | 1.22 (0.82 – 1.81) | 0.336 |
| Loose, 3 MQ doses | 1106 | 14.6 | Reference |  |
| Co-blister, 3 MQ doses | 110 | 22.7 | 1.49 (0.44 – 5.06) | 0.520 |
| Supervision |  |  |  |  |
| Partial | 359 | 17.8 | 0.70 (0.27 - 1.83) | 0.464 |
| Full | 3439 | 26.5 | Reference |  |
| Age (years) | 3798 | 25.7 | 0.98 (0.97 - 0.99) | <0.001 |
| Age group |  |  |  |  |
| < 1 year | 57 | 87.7 | 14.64 (5.66 - 37.85) | <0.001 |
| 1 to <5 years | 553 | 68.4 | 5.80 (4.19 - 8.03) | <0.001 |
| 5 to <12 years | 549 | 33.9 | 2.03 (1.55 - 2.65) | <0.001 |
| ≥ 12 years | 2639 | 13.7 | Reference |  |
| Underweight (<5 years of age)^2^ |  |  |  |  |
| Yes | 112 | 75.0 | 1.25 (0.72 – 2.18) | 0.423 |
| No | 496 | 69.2 | Reference |  |
| Sex |  |  |  |  |
| Male | 2328 | 21.3 | 0.57 (0.46 - 0.69) | <0.001 |
| Female | 1470 | 32.7 | Reference |  |
| Temperature (C ) | 3631 | 25.8 | 1.23 (1.12 - 1.35) | <0.001 |
| Fever or history of fever^6^ |  |  |  |  |
| Yes | 2387 | 28.8 | 1.27 (1.01 - 1.59) | 0.044 |
| No | 1263 | 20.0 | Reference |  |
| Log10 Parasitaemia (/µL) | 3798 | 25.7 | 1.64 (1.43 - 1.88) | <0.001 |
| Hyperparasitaemia ^7^ |  |  |  |  |
| Yes | 342 | 41.5 | 1.89 (1.37 - 2.59) | <0.001 |
| No | 3456 | 24.1 | Reference |  |
| Gametocytes on enrolment |  |  |  |  |
| Yes | 319 | 41.4 | 1.61 (1.16 - 2.22) | 0.004 |
| No | 3456 | 24.4 | Reference |  |
| Transmission Intensity^8^ |  |  |  |  |
| High | 60 | 75.0 | 4.03 (0.67 - 24.43) | 0.129 |
| Moderate | 493 | 36.1 | 1.41 (0.58 - 3.43) | 0.443 |
| Low | 3245 | 23.2 | Reference |  |
| Region |  |  |  |  |
| Africa | 705 | 62.8 | Reference |  |
| Asia | 2792 | 18.1 | 0.22 (0.15 – 0.32) | <0.001 |
| South America | 301 | 9.3 | 0.12 (0.05 – 0.27) | <0.001 |

^1^comparison between Fixed, 3 MQ doses and Loose, 2 MQ doses: OR 1.70, 95%CI 1.11 - 2.61, p= 0.014; comparison between Fixed, 3 MQ doses and combined Loose, 3 MQ doses + Coblister: OR 1.99, 95%CI 1.22 - 3.27, p= 0.006; ^2^Underweight defined in children <5 years of age, as weight-for age z-score <–2; ^3^Ht converted to Hb using the formula $Hematocrit =5\cdot62+2\cdot60 \times Haemoglobin$ (Lee, 2008); ^4^Severe anaemia defined as haemoglobin <7 g/dL or haematocrit<20%; ^5^Moderate anaemia as 7 g/dL ≤haemoglobin <10 g/dL or 20%≤haematocrit<30%; ^6^Defined as temperature >37·5°C or reported history of fever; ^7^Defined as parasitaemia >10^5^/μL; ^8^Malaria transmission intensity defined from estimates of *Plasmodium falciparum* prevalence rate (PfPR) according to enrolment year and location, assuming low transmission for study sites with a PfPR <0·10, moderate transmission if PfPR 0·10 to <0·40, and high transmission if PfPR ≥0·40.

Anaemia at baseline, young age, female sex, high temperature, high baseline parasitaemia, gametocyte carriage, transmission intensity and region were also associated with the increased risk of anaemia on day 7. No association was observed with artemisinin resistance level. In the sensitivity analysis, exclusion of study ID 7, the only study included in this analysis in which no MQ dose was given at 48 hours, gave smaller non-significant dose effect, with a much wider confidence interval. For this dataset, all other MQ dose variables were also non-significant. Risk of anaemia was significantly smaller for study ID7 (i.e. for no MQ at 48h) compared to other studies which gave MQ at 48h (AOR 0.36, 95%CI 1.06 – 1.19, p=0.003)

## Supplementary Table 16. Serious Adverse Events and neurological adverse events in patients treated with AS-MQ

| **Study number** | **CNS AE or other SAE** | **Comments including AS-MQ formulation and brand** |
| --- | --- | --- |
| 1 | Child 1-13y; seizures (n=1)  Child 1-13y; fractured radius requiring hospitalization (n=1) | PMID 20361381  Both received co-blister tablet formulation (Artequin™  300/750)  Both assessed as unrelated to study drugs |
| 2 | None |  |
| 3 | None |  |
| 4 | None |  |
| 5 | None |  |
| 6 | None |  |
| 7 | None |  |
| 8 | None |  |
| 9 | None |  |
| 10 | None |  |
| 11 | 30yM; Toxic metabolic encephalopathy (n=1)  41yM; weakness, anxiety, transient arrythmia (n=1)  6yM; palpitations and chest pain (n=1)  Adult female; anxiety and insomnia requiring medication | PMID 17971864  Regimen: MQ= 8 mg/kg/day (LariamTM, Hoffman-La Roche Co., Basel,  Switzerland) and AS = 4 mg/kg/day (Guilin Pharmaceutical  Factory, China)  Reactions necessitated stopping AS-MQ for 3 male patients.  Not specified if female patient received AS-MQ or DHA-piperaquine |
| 12 | None |  |
| 13 | None |  |
| 14 | None |  |
| 15 | None |  |
| 16 | ARDS on day 4 (n=1) | PMID 22232454  AS-MQ = loose combination  of Plasmotrim and Mephaquin, Mepha, Aesch, Switzerland); artesunate once daily for 3 days with mefloquine on Days 2 and 3.  Assessed as unrelated to AS-MQ |
| 17 | Seizures (n=2)  Cerebral malaria (n=1) | PMID 22475593  Artesunate (Guilin Pharmaceutical Co. China), 4 mg/kg/day for 3 days plus mefloquine (Lariam; Roche), 15 mg/kg on day 1 and 10 mg/kg on day 2  Seizures assessed as related to AS-MQ  AS-MQ treatment stopped for patient with severe malaria |
| 18 | Adult men; severe nightmares and hallucinations (n=2)  8yF; hallucinations and anxiety | PMID 15486837  Artesunate (Guilin Pharmaceutical  Co., China), 4 mg/kg/day for 3 days (days  0–2), plus mefloquine (Lariam; Roche), 15 mg/kg on day 1 and 10 mg/kg on day 2  Assessed as related to AS-MQ |
| 19 | 13yM; death with abdominal symptoms ?cause 6 days after presentation  3yM; anaemia requiring blood transfusion  5yM; seizure (?febrile convulsion) 50yF; jaundice, bleeding, hepatosplenomegaly 49 days after presentation (?alcoholic liver disease) | PMID 16028147  AS + MEF (Artequin™; Mepha Ltd., Basel, Switzerland)  Assessed as unrelated to AS-MQ |
| 20 | 17yM; agitation, insomnia, severe nightmares, delirium, anxiety on day 3. | PMID 16903879  Artesunate, Mepha, Aesch-  Basel Switzerland), 4 mg/kg/day for 3 days  plus mefloquine (Mephaquin, Mepha, Aesch-  Basel Switzerland) 15 mg base/kg on day 1 and 10 mg  base/kg on day 2,  Relationship to AS_MQ not specified but likely related |
| 21 | None |  |
| 22 | 40yF; severe anaemia  Anaemia (n=1) on day 8  Seizure (n=1) on day 1  Encephalitis (n=1) on day 16-31 | PMID 22818552;20689583  Artesunate (Guilin  Pharmaceutical Factory,  China) or Arsumax (Sanofi-Aventis, Gentilly-France) 4 mg/kg/day for 3 days once daily, and mefloquine 25 mg base/kg, Lariam (Roche, Basel-Switzerland) or Mequin (Atlantic Laboratories, Bangkok-Thailand).  One anaemia case assessed as unrelated to AS-MQ. Other 3 SAEs assessed as related to AS-MQ |
| 23 | 8 SAEs (during first period of follow up)  Infection (n=7)  Anaemia, severe malaria, sepsis (n=1)  Seizures (incl. febrile seizures) (n=6) | PMID 27430374  Patients with SAEs received fixed-dose tablets (Far-Manguinhos, Rio de Janeiro, Brazil;  2 SAEs led to drug discontinuation; 1 assessed as related to AS-MQ |
| 24 | None |  |
| 25 | Adults; severe malaria (n=2) | PMID 20547795  Artesunate (Arsumax; Sanofi-Aventis) and mefloquine (Eloquine; Medochemie)  Assessed as unrelated to AS-MQ |
| 26 | 12 SAEs during 9 weeks  of follow-up; all required hospitalization. Child; repeated  vomiting requiring admission for intravenous hydration (n=1)  Measles (n=1)  Bacterial sepsis (n=1)  Renal colic (n=1)  Pyelonephritis (n=1)  Diarrhoea (n=1)  Vomiting (n=2)  Dehydration (n=1)  Relapse of pre-existing  nephrotic syndrome (n=1)  Palpitations (n=1)  Pyrexia of unknown origin(n=1) | PMID 17054744Artequin paediatric (Mepha) granules of 50 mg of artesunate and 125 mg of mefloquine as a  fixed-dose combination  Repeated vomiting assessed as related to AS_MQ |
| 27 | None |  |
| 28 | None |  |
| 29 | None |  |
| 30 | None |  |
| 31 | Cutaneous hypersensitivity reaction (n=1) | PMID 11132386  Artesunate (Guilin Pharmaceutical Factory no.1, Guilin, China) and Mefloquine (Lariam@. Hoffman-La-Roche, Basel, Switzerland)Assessed related to AS or MQ and medicine discontinued |

Note: AE: adverse event; SAE serious adverse event; PMID= PubMed ID

## Supplementary Table 17. Proportion of patients with vomiting, days 0-3, by study

| Study  ID |  | Day 0 | | Day 1 | | Day 2 | | Day 3 | |
| --- | --- | --- | --- | --- | --- | --- | --- | --- | --- |
|  |  | N | % | N | % | N | % | N | % |
| 4 | BXTRR | 75 | 9.3 | 74 | 0.0 | 75 | 0.0 | 73 | 0.0 |
| 5 | BYDYQ | 163 | 2.5 | 163 | 0.0 | 162 | 0.6 | 162 | 0.0 |
| 7 | CUDNY | 226 | 28.3 | 224 | 22.3 | 224 | 7.6 | 216 | 1.9 |
| 11 | FFNAU | 258 | 9.7 | 258 | 13.6 | 258 | 10.1 | 258 | 8.1 |
| 13 | GHGRR | 72 | 16.7 | 72 | 4.2 | 72 | 4.2 | 72 | 4.2 |
| 16 | HCEMT | 52 | 0.0 | 52 | 11.5 | 52 | 11.5 | 52 | 1.9 |
| 17 | HGNAF | 416 | 20.0 | 413 | 2.2 | 416 | 2.4 | 414 | 0.5 |
| 21 | NBSAE | 194 | 38.7 | 194 | 4.6 | 194 | 0.5 | 194 | 1.0 |
| 23 | RMRNH | 459 | 14.8 | 459 | 8.5 | 459 | 2.6 | 459 | 1.3 |
| 25 | SUGYV | 47 | 57.4 | 47 | 34.0 | 47 | 27.7 | 47 | 27.7 |
| 26 | SZRDK | 496 | 23.2 | 493 | 18.1 | 487 | 7.4 | 164 | 6.1 |
| 28 | XEKED | 157 | 19.1 | 156 | 7.1 | 156 | 3.2 | 155 | 0.6 |

## Supplementary Table 18. Proportion of patients with diarrhoea, days 0-3, by study

| Study  ID |  | Day 0 | | Day 1 | | Day 2 | | Day 3 | |
| --- | --- | --- | --- | --- | --- | --- | --- | --- | --- |
|  |  | N | % | N | % | N | % | N | % |
| 4 | BXTRR | 75 | 4.0 | 74 | 6.8 | 75 | 1.3 | 73 | 0.0 |
| 5 | BYDYQ | 163 | 0.6 | 163 | 0.0 | 162 | 0.0 | 162 | 0.6 |
| 7 | CUDNY | 226 | 18.1 | 223 | 15.2 | 224 | 6.7 | 216 | 3.2 |
| 11 | FFNAU | 258 | 1.6 | 258 | 9.7 | 258 | 8.9 | 258 | 4.7 |
| 13 | GHGRR | 72 | 15.3 | 72 | 12.5 | 72 | 9.7 | 72 | 1.4 |
| 16 | HCEMT | 52 | 0.0 | 52 | 9.6 | 52 | 1.9 | 52 | 1.9 |
| 21 | NBSAE | 194 | 8.8 | 194 | 0.5 | 194 | 0.0 | 194 | 0.5 |
| 23 | RMRNH | 459 | 10.0 | 459 | 6.1 | 459 | 2.2 | 459 | 1.7 |
| 25 | SUGYV | 47 | 34.0 | 47 | 27.7 | 47 | 27.7 | 47 | 29.8 |
| 26 | SZRDK | 496 | 3.8 | 493 | 5.5 | 487 | 4.1 | 164 | 1.8 |

## Supplementary Table 19. Univariable analysis of vomiting and diarrhoea on day 1-3.

Poisson regression models with random intercept for study-site were fitted. Models adjusted for baseline symptoms.

|  | Vomiting | | | | | | | Diarrhoea | | | | | |
| --- | --- | --- | --- | --- | --- | --- | --- | --- | --- | --- | --- | --- | --- |
|  | N | %^1^ | | IRR (95% CI) | | P-value | | N | | %^1^ | | IRR (95%CI) | P-value |
| Total MQ dose (mg/kg) | 2264 | 15.6 | | 1.03 (1.01 - 1.05) | | 0.005 | | 1693 | | 11.5 | | 0.95 (0.92 - 0.98) | 0.004 |
| Number of MQ doses |  |  | |  | |  | |  | |  | |  |  |
| Three | 1753 | 12.4 | | 0.77 (0.58 - 1.04) | | 0.084 | | 1182 | | 10.4 | | 1.19 (0.80 - 1.76) | 0.392 |
| Two | 511 | 26.4 | | Reference | |  | | 511 | | 13.9 | | Reference |  |
| MQ dose (mg/kg) at 24h | 2264 | 15.6 | | 1.05 (1.02 - 1.09) | | 0.003 | | 1693 | | 11.5 | | 0.95 (0.91 - 1.00) | 0.046 |
| Total MQ dose (mg/kg) over first 24h | 2264 | 15.6 | | 1.04 (1.01 - 1.07) | | 0.016 | | 1693 | | 11.5 | | 0.94 (0.90 - 0.99) | 0.010 |
| Maximum MQ dose (mg/kg) | 2264 | 15.6 | | 1.06 (1.02 - 1.09) | | <0.001 | | 1693 | | 11.5 | | 0.95 (0.90 - 0.99) | 0.024 |
| Total AS dose (mg/kg) | 2264 | 15.6 | | 1.06 (1.02 - 1.11) | | 0.004 | | 1693 | | 11.5 | | 0.91 (0.86 - 0.97) | 0.006 |
| Drug formulation^2^ |  |  | |  | |  | |  | |  | |  |  |
| Fixed, 3 MQ doses | 885 | 14.8 | | 2.85 (1.22 - 6.65) | | 0.016 | | 730 | | 10.8 | | 0.64 (0.14 - 2.82) | 0.553 |
| Loose, 2 MQ doses | 511 | 26.4 | | 3.57 (1.52 - 8.40) | | 0.004 | | 511 | | 13.9 | | 0.55 (0.12 - 2.48) | 0.439 |
| Loose, 3 MQ doses | 674 | 11.3 | | Reference | |  | | 258 | | 16.3 | | Reference |  |
| Co-blister, 3 MQ doses | 194 | 5.7 | | 0.58 (0.07 - 5.15) | | 0.625 | | 194 | | 1.0 | | 0.03 (0.03 - 0.39) | 0.007 |
| Supervision |  |  | |  | |  | |  | |  | |  |  |
| Partial | 494 | 22.5 | | 1.93 (0.57 - 6.55) | | 0.294 | | 494 | | 7.7 | | 0.66 (0.25 - 1.76) | 0.402 |
| Full | 1770 | 13.7 | | Reference | |  | | 1199 | | 13.0 | | Reference | . |
| Age (years) | 2264 | 15.6 | | 0.99 (0.98 - 1.00) | | 0.007 | | 1693 | | 11.5 | | 1.01 (1.00 - 1.02) | 0.075 |
| Age group |  |  | |  | |  | |  | |  | |  |  |
| < 1 year | 34 | 29.4 | | 0.89 (0.42 - 1.88) | | 0.759 | | 32 | | 40.6 | | 2.47 (1.11 - 5.46) | 0.026 |
| 1 to <5 years | 284 | 19.7 | | 1.08 (0.69 - 1.68) | | 0.744 | | 219 | | 11.9 | | 0.94 (0.47 - 1.88) | 0.865 |
| 5 to <12 years | 415 | 11.8 | | 0.78 (0.57 - 1.07) | | 0.120 | | 273 | | 3.7 | | 0.52 (0.30 - 0.91) | 0.021 |
| ≥ 12 years | 1531 | 15.5 | | Reference | |  | | 1169 | | 12.4 | | Reference |  |
| Underweight (<5 years of age)^3^ |  | |  | |  | |  | |  | |  |  |  |
| Yes | 47 | | 14.9 | | 0.83 (0.42 – 1.61) | | 0.573 | | 36 | | 16.7 | 0.80 (0.37-1.72) | 0.564 |
| No | 270 | | 21.9 | | Reference | |  | | 215 | | 15.3 | Reference |  |
| Sex |  |  | |  | |  | |  | |  | |  |  |
| Male | 1334 | 13.0 | | 0.83 (0.68 - 1.01) | | 0.067 | | 912 | | 12.1 | | 0.86 (0.66 - 1.12) | 0.274 |
| Female | 930 | 19.2 | | Reference | |  | | 781 | | 10.8 | | Reference |  |
| Haemoglobin^4^ | 2086 | 16.5 | | 1.05 (1.00 - 1.10) | | 0.047 | | 1681 | | 11.4 | | 1.05 (0.99 - 1.11) | 0.130 |
| Moderate/Severe anaemia^5^ |  |  | |  | |  | |  | |  | |  |  |
| Yes | 344 | 16.9 | | 0.89 (0.67 - 1.19) | | 0.444 | | 234 | | 14.1 | | 0.87 (0.60 - 1.26) | 0.464 |
| No | 1742 | 16.5 | | Reference | |  | | 1447 | | 11.0 | | Reference |  |
| Severe anaemia^6^ |  |  | |  | |  | |  | |  | |  |  |
| Yes | 35 | 40.0 | | 2.65 (1.60 - 4.40) | | <0.001 | | 22 | | 31.8 | | 1.57 (0.76 - 3.25) | 0.224 |
| No | 2051 | 16.1 | | Reference | |  | | 1659 | | 11.2 | | Reference |  |
| Temperature (C ) | 2064 | 16.7 | | 1.08 (0.99 - 1.18) | | 0.081 | | 1691 | | 11.5 | | 1.07 (0.96 - 1.19) | 0.244 |
| Fever or history of fever^7^ |  |  | |  | |  | |  | |  | |  |  |
| Yes | 1753 | 17.3 | | 1.12 (0.82 - 1.53) | | 0.491 | | 1480 | | 11.0 | | 1.17 (0.81 - 1.70) | 0.392 |
| No | 313 | 13.7 | | Reference | |  | | 213 | | 14.6 | | Reference |  |
| Log10 Parasitaemia (/µL) | 2264 | 15.6 | | 0.90 (0.80 - 1.02) | | 0.093 | | 1693 | | 11.5 | | 0.97 (0.84 - 1.12) | 0.656 |
| Hyperparasitaemia^8^ |  |  | |  | |  | |  | |  | |  |  |
| Yes | 131 | 29.0 | | 1.01 (0.73 - 1.41) | | 0.944 | | 129 | | 14.0 | | 0.88 (0.58 - 1.32) | 0.532 |
| No | 2133 | 14.8 | | Reference | |  | | 1564 | | 11.3 | | Reference |  |
| Gametocytes on enrolment |  |  | |  | |  | |  | |  | |  |  |
| Yes | 244 | 11.1 | | 0.78 (0.55 - 1.10) | | 0.149 | | 189 | | 9.5 | | 0.84 (0.57 - 1.23) | 0.364 |
| No | 2020 | 16.1 | | Reference | |  | | 1504 | | 11.7 | | Reference |  |
| Transmission Intensity^9^ |  |  | |  | |  | |  | |  | |  |  |
| High | 207 | 6.3 | | 0.33 (0.05 - 2.17) | | 0.249 | | 207 | | 1.9 | | 0.27 (0.05 - 1.36) | 0.113 |
| Moderate | 403 | 23.8 | | 1.07 (0.34 - 3.34) | | 0.907 | | 351 | | 18.2 | | 1.31 (0.49 - 3.50) | 0.596 |
| Low | 1654 | 14.8 | | Reference | |  | | 1135 | | 11.1 | | Reference |  |
| Region |  |  | |  | |  | |  | |  | |  |  |
| Africa | 576 | 13.4 | | 0.91 (0.36 - 2.33) | | 0.852 | | 343 | | 10.5 | | 0.87 (0.36 - 2.13) | 0.758 |
| South America | 439 | 16.6 | | 2.10 (0.48 - 9.09) | | 0.322 | | 439 | | 11.4 | | 1.46 (0.45 - 4.71) | 0.529 |
| Asia | 1249 | 16.3 | | Reference | |  | | 911 | | 11.9 | | Reference |  |

^1^ % of patients with symptoms over Days 1-3 ^2^comparison between Fixed, 3 MQ doses and Loose, 2 MQ doses: p =0.153 for vomiting, and p=0.445 for diarrhoea; ^3^Underweight defined in children <5 years of age, as weight-for age z-score <–2;^4^Ht converted to Hb using the formula $Hematocrit =5\cdot62+2\cdot60 \times Haemoglobin$ (Lee, 2008); ^5^Severe anaemia defined as haemoglobin <7 g/dL or haematocrit<20%; ^6^Moderate anaemia as 7 g/dL ≤haemoglobin <10 g/dL or 20%≤haematocrit<30%; ^7^Defined as temperature >37·5°C or reported history of fever; ^8^Defined as parasitaemia >10^5^/μL; ^9^Malaria transmission intensity defined from estimates of *Plasmodium falciparum* prevalence rate (PfPR) according to enrolment year and location, assuming low transmission for study sites with a PfPR <0·10, moderate transmission if PfPR 0·10 to <0·40, and high transmission if PfPR ≥0·40

## Supplementary Table 20. Univariable analysis of vomiting and diarrhoea during 24h post dose.

Logistic regression models with random intercept for patient and fixed effect for study were fitted. Models adjusted for baseline symptoms.

|  | Vomiting | | | | | | | Diarrhoea | | | | | |
| --- | --- | --- | --- | --- | --- | --- | --- | --- | --- | --- | --- | --- | --- |
|  | N | %^1^ | | OR (95% CI) | | P-value | | N | | %^1^ | | IRR (95%CI) | P-value |
| Total MQ dose (mg/kg) in the last 24h | 6208 | 7.4 | | 1.05 (1.03 - 1.07) | | <0.001 | | 4722 | | 6.0 | | 1.05 (1.02 - 1.08) | <0.001 |
| Maximum MQ dose (mg/kg) in the last 24h | 6208 | 7.4 | | 1.02 (0.99 - 1.04) | | 0.277 | | 4722 | | 6.0 | | 1.03 (0.98 - 1.07) | 0.205 |
| Total AS dose (mg/kg) in the last 24h | 6208 | 7.4 | | 0.80 (0.67 - 0.96) | | 0.016 | | 4722 | | 6.0 | | 0.47 (0.35 - 0.62) | <0.001 |
| Maximum AS dose (mg/kg) in the last 24h | 6208 | 7.4 | | 0.89 (0.75 - 1.07) | | 0.219 | | 4722 | | 6.0 | | 0.48 (0.35 - 0.66) | <0.001 |
| Number of MQ doses |  |  | |  | |  | |  | |  | |  |  |
| Three | 5917 | 5.1 | | 0.77 (0.49 - 1.20) | | 0.246 | | 4337 | | 9.4 | | 1.29 (0.79 - 2.11) | 0.312 |
| Two | 1796 | 10.8 | | Reference | |  | | 1793 | | 6.9 | | Reference |  |
| Drug formulation^2^ |  |  | |  | |  | |  | |  | |  |  |
| Fixed, 3 MQ doses | 2860 | 6.4 | | 1.78 (0.82- 3.83) | | 0.142 | | 2529 | | 5.7 | | 0.50 (0.32 - 0.79) | 0.003 |
| Loose, 2 MQ doses | 1796 | 10.8 | | 2.32 (0.95 – 5.64) | | 0.064 | | 1793 | | 6.9 | | 0.39 (0.24 - 0.64) | <0.001 |
| Loose, 3 MQ doses | 2281 | 4.8 | | Reference | |  | | 1032 | | 6.5 | | Reference |  |
| Co-blister, 3 MQ doses | 776 | 1.5 | | 0.75 (0.33 – 1.70) | | 0.493 | | 776 | | 25.3 | | 5.09 (3.53-7.32) | <0.001 |
| Supervision |  |  | |  | |  | |  | |  | |  |  |
| Partial | 1581 | 8.8 | | 3.90 (2.05 - 7.39) | | <0.001 | | 1581 | | 3.5 | | 0.18 (0.10 - 0.31) | <0.001 |
| Full | 6132 | 5.9 | | Reference | |  | | 4549 | | 10.4 | | Reference |  |
| Age (years) | 7713 | 6.5 | | 0.98 (0.97 - 0.99) | | 0.004 | | 6130 | | 8.6 | | 1.01 (1.00 - 1.02) | 0.041 |
| Age group |  |  | |  | |  | |  | |  | |  |  |
| < 1 year | 105 | 10.5 | | 0.80 (0.25 - 2.55) | | 0.709 | | 100 | | 22.0 | | 2.45 (1.04 - 5.79) | 0.041 |
| 1 to <5 years | 1005 | 7.7 | | 1.08 (0.56 - 2.09) | | 0.824 | | 750 | | 15.1 | | 0.75 (0.47 - 1.21) | 0.238 |
| 5 to <12 years | 1556 | 3.7 | | 0.83 (0.54 - 1.27) | | 0.385 | | 1045 | | 11.2 | | 0.74 (0.50 - 1.08) | 0.117 |
| ≥ 12 years | 5047 | 7.0 | | Reference | |  | | 4235 | | 6.6 | | Reference |  |
| Underweight (<5 years of age)^3^ |  | |  | |  | |  | |  | |  |  |  |
| Yes | 159 | | 6.3 | | 0.62 (0.26-1.51) | | 0.295 | | 117 | | 16.2 | 0.92 (0.53-1.61) | 0.771 |
| No | 936 | | 8.33 | |  | |  | | 722 | | 15.4 |  |  |
| Sex |  |  | |  | |  | |  | |  | |  |  |
| Male | 4474 | 5.8 | | 0.81 (0.61 - 1.08) | | 0.155 | | 3380 | | 8.8 | | 0.93 (0.73 - 1.19) | 0.571 |
| Female | 3239 | 7.4 | | Reference | |  | | 2750 | | 8.4 | | Reference |  |
| Haemoglobin^4^ | 7168 | 6.8 | | 1.06 (0.99 - 1.13) | | 0.108 | | 6093 | | 8.6 | | 1.04 (0.98 - 1.10) | 0.222 |
| Moderate/Severe anaemia^5^ |  |  | |  | |  | |  | |  | |  |  |
| Yes | 1188 | 6.7 | | 0.88 (0.58 - 1.32) | | 0.531 | | 806 | | 10.7 | | 0.85 (0.60 - 1.21) | 0.370 |
| No | 5980 | 6.9 | | Reference | |  | | 5287 | | 8.3 | | Reference |  |
| Severe anaemia^6^ |  |  | |  | |  | |  | |  | |  |  |
| Yes | 119 | 16.8 | | 4.30 (1.90 - 9.73) | | <0.001 | | 71 | | 19.7 | | 1.75 (0.79 - 3.87) | 0.164 |
| No | 7049 | 6.7 | | Reference | |  | | 6022 | | 8.5 | | Reference |  |
| Temperature (C ) | 7113 | 6.9 | | 1.17 (1.03 - 1.33) | | 0.019 | | 6124 | | 8.7 | | 1.06 (0.94 - 1.19) | 0.320 |
| Fever or history of fever^7^ |  |  | |  | |  | |  | |  | |  |  |
| Yes | 6082 | 7.2 | | 1.26 (0.81 - 1.96) | | 0.309 | | 5346 | | 9.2 | | 1.42 (0.93 - 2.16) | 0.104 |
| No | 1037 | 5.2 | | Reference | |  | | 784 | | 5.1 | | Reference |  |
| Log10 Parasitaemia (/µL) | 7713 | 6.5 | | 0.87 (0.72 - 1.03) | | 0.111 | | 6130 | | 8.6 | | 0.92 (0.79 - 1.09) | 0.342 |
| Hyperparasitaemia^8^ |  |  | |  | |  | |  | |  | |  |  |
| Yes | 447 | 10.7 | | 1.04 (0.63 - 1.72) | | 0.865 | | 443 | | 10.2 | | 0.84 (0.55 - 1.29) | 0.433 |
| No | 7266 | 6.2 | | Reference | |  | | 5687 | | 8.5 | | Reference |  |
| Gametocytes on enrolment |  |  | |  | |  | |  | |  | |  |  |
| Yes | 861 | 4.9 | | 0.73 (0.45 - 1.19) | | 0.212 | | 719 | | 5.3 | | 0.83 (0.53 - 1.31) | 0.428 |
| No | 6852 | 6.7 | | Reference | |  | | 5411 | | 9.1 | | Reference |  |
| Transmission Intensity^9^ |  |  | |  | |  | |  | |  | |  |  |
| High | 815 | 1.7 | | 0.22 (0.03 - 1.48) | | 0.119 | | 815 | | 24.4 | | 0.59 (0.13 - 2.72) | 0.495 |
| Moderate | 1471 | 9.4 | | 1.25 (0.60 - 2.62) | | 0.554 | | 1321 | | 8.2 | | 1.41 (0.66 - 3.03) | 0.373 |
| Low | 5427 | 6.4 | | Reference | |  | | 3994 | | 5.6 | | Reference |  |
| Region |  |  | |  | |  | |  | |  | |  |  |
| Africa | 2079 | 4.6 | | 0.69 (0.20 - 2.41) | | 0.561 | | 1236 | | 20.6 | | 2.32 (1.46 - 3.68) | <0.001 |
| South America | 1735 | 6.0 | | 22.27 (4.19 - 118.34) | | <0.001 | | 1733 | | 4.3 | | 5.42 (2.07 - 14.18) | <0.001 |
| Asia | 3899 | 7.7 | | Reference | |  | | 3161 | | 6.3 | | Reference |  |

^1^ % of patients with symptoms over Days 1-3 ^2^comparison between Fixed, 3 MQ doses and Loose, 2 MQ doses: p =0.153 for vomiting, and p=0.445 for diarrhoea; ^3^Underweight defined in children <5 years of age, as weight-for age z-score <–2;^4^Ht converted to Hb using the formula $Hematocrit =5\cdot62+2\cdot60 \times Haemoglobin$ (Lee, 2008); ^5^Severe anaemia defined as haemoglobin <7 g/dL or haematocrit<20%; ^6^Moderate anaemia as 7 g/dL ≤haemoglobin <10 g/dL or 20%≤haematocrit<30%; ^7^Defined as temperature >37·5°C or reported history of fever; ^8^Defined as parasitaemia >10^5^/μL; ^9^Malaria transmission intensity defined from estimates of *Plasmodium falciparum* prevalence rate (PfPR) according to enrolment year and location, assuming low transmission for study sites with a PfPR <0·10, moderate transmission if PfPR 0·10 to <0·40, and high transmission if PfPR ≥0·40

# 4. Main Analysis Supplementary Figures

## Supplementary Figure 1. Map of study sites

## Supplementary Figure 2. Artesunate (panel A) and mefloquine (panel B) doses administered by weight, by study

B

A

## Supplementary Figure 3. Artesunate (panel A) and mefloquine (panel B) doses administered, by treatment formulation

## Supplementary Figure 4. Artesunate (A) and mefloquine (B) doses administered, by weight

B

A

## Supplementary Figure 5. Day 2 positivity by study

.

## Supplementary Figure 6. Day3 parasite positivity by study

## Supplementary Figure 7. MQ dose by 24H effect for positivity on Day 2 by study

## Supplementary Figure 8. MQ dose effect by study for parasite positivity on Day 3 in Asia

## Supplementary Figure 9. Mefloquine dose (mg/kg) received at 24h by region. Data in Asia and South America include 2-dose regimens. Only 3-dose regimens were studied in Africa

## Supplementary Figure 10. Recrudescence by study – outcome day 28,42,63

## Supplementary Figure 11. Kaplan-Meier estimates of proportion of patients with recrudescence by region and artemisinin resistance level

ARTr – Artemisinin resistance level

## Supplementary Figure 12. MQ dose effect for recrudescence by study

## Supplementary Figure 13. Reinfection by study

B

## Supplementary Figure 14. Kaplan-Meier estimate of proportion of patients with reinfection, by transmission intensity

TIA - Transmission Intensity Area

## Supplementary Figure 15: MQ dose effect for reinfection by study

# 5. Sensitivity analysis

Early Parasitological Response

These findings were confirmed in the sensitivity analysis (Additional File 1) for day 2 positivity. For day 3, one study (ID 29, N=855/6028 patients, n =131/254 positive slides) had impact on the findings, with a slightly reduced dose effect and wider non-significant confidence intervals including 1 observed after its exclusion, while the effect of excluding the MQ manufacturer was small and only affected confidence intervals due to the reduced sample size (Additional File 1: Supplementary Table 18). While slide positivity on day 2 or 3 among patients receiving fixed or loose 3-dose formulations were not significantly different in Asia (Table 3), in Africa and South America, patients treated with the fixed combination had higher risk of a parasite positive slide on day 2 (Africa AOR 2.66, 95%CI 1.16-6.11, p=0.021; South America AOR 2.49, 95%CI 0.94 -6.63, p=0.068 adjusted for baseline parasitaemia) compared to those treated with 3-day loose combinations (including co-blister in Africa).

In studies from Africa and South America, no other variables were independently associated with parasite positivity, so no multivariable models were built.

## Supplementary Figure 16. Estimates (95% CI) of the dose effect (as in multivariable analysis tables) after exclusion of one study at a time. Study ID of the excluded study is shown on the x-axis. All – denotes estimates from the model fitted on all data. Estimates are OR, HR, RR or IRR

## Supplementary Figure 17. Estimates (95% CI) for the comparison between different MQ regimens after exclusion of one study at a time. The comparison group is 3-dose loose regimen. Study ID of the excluded study is shown on the x-axis. All – denotes estimates from the model fitted on all data. Estimates are OR, HR, RR or IRR

## Supplementary Figure 18. Estimates (95% CI) of the dose effect ( as in multivariable analysis tables) after exclusion of one manufacturer at a time, for supervised data , and for studies using at least 3 markers for PCR genotyping. All – denotes estimates from the model fitted on all data. Estimates are OR, HR, RR or IRR

## Supplementary Figure 19. Estimates (95% CI) for the comparison between different MQ regimens after exclusion of one manufacturer at a time, for supervised data only, and for studies using at least 3 markers for PCR genotyping. All – denotes estimates from the model fitted on all data. Estimates are OR, HR, RR or IRR

# 5 Supplementary References

1.	WorldWide Antimalarial Resistance Network. Clinical Module: Data Management and Statistical Analysis Plan Version 1.2. 2012 [Available from: <http://www.wwarn.org/sites/default/files/ClinicalDMSAP.pdf>.

2.	Gething PW, Patil AP, Smith DL, Guerra CA, Elyazar IR, Johnston GL, et al. A new world malaria map: Plasmodium falciparum endemicity in 2010. Malaria journal. 2011;10:378.

3.	World Health Organization. Malaria surveillance, monitoring & evaluation: a reference manual 2018 [Available from: <https://apps.who.int/iris/bitstream/handle/10665/272284/9789241565578-eng.pdf>.

4.	de Onis M, Garza C, Victora CG, Onyango AW, Frongillo EA, Martines J. The WHO Multicentre Growth Reference Study: planning, study design, and methodology. Food and nutrition bulletin. 2004;25(1 Suppl):S15-26.

5.	WHO Multicentre Growth Reference Study Group. WHO Child Growth Standards: Length/height-for-age, weight-for-age, weight-for-length, weight-for-height and body mass index-for-age: Methods and development. Geneva World Health Organization. 2006:312.

6.	Lee SJ, Stepniewska K, Anstey N, Ashley E, Barnes K, Binh TQ, et al. The relationship between the haemoglobin concentration and the haematocrit in Plasmodium falciparum malaria. Malaria journal. 2008;7:149.

7.	Collett D. Modelling Survival Data in Medical Research. 3rd ed: CRC Press; 2015.

8.	Royston P, Altman DG. Regression using fractional polynomials of continuous covariates: parsimonious parametric modelling. Applied Statistics. 1994;43:429-67.
